# Biochemical and Structural Characterization of Two-domain Glycoside Hydrolase PgaB from *Serratia marcescens* and Its Application for *S. aureus* Biofilm Degradation

**DOI:** 10.1021/acsinfecdis.6c00086

**Published:** 2026-06-15

**Authors:** Amanda Freitas Cruz, Pedro Ricardo Vieira Hamann, Francisco Eduardo Gontijo Guimaraes, Andrei Nicoli Gebieluca Dabul, Ruth Celestina Condori Mamani, Marcos Pileggi, Mario de Oliveira Neto, Matheus Rodrigues Sauda, Guilherme Targino Valente, Tsutomu Matsui, Thomas M. Weiss, Evandro A. Araújo, Igor Polikarpov

**Affiliations:** 1 Instituto de Física de São Carlos, Universidade de São Paulo, Avenida Trabalhador São-carlense 400, São Carlos, SP 13566-590, Brazil; 2 School of Pharmaceutical Sciences, São Paulo State University, Rodovia Araraquara-Jáu, km 1, Araraquara, SP 14800-903, Brazil; 3 Environmental Microbiology Laboratory, Life Sciences and Health Institute, Structural and Molecular Biology, and Genetics Department, 67883 Ponta Grossa State University, Ponta Grossa 84030-900, Brazil; 4 Institute of Biosciences, 42512 Sao Paulo State University, District of Rubiao Jr., Botucatu, SP 18618-970, Brazil; 5 Laboratory of Applied Biotechnology, São Paulo State University, Botucatu 18618-687, Brazil; 6 Clinical Hospital of Medical School of Botucatu, Botucatu 18618-687, Brazil; 7 Stanford Synchrotron Radiation Lightsource, SLAC National Accelerator Laboratory, 2575 Sand Hill Rd, Menlo Park, California 94025, United States; 8 Brazilian Synchrotron Light Laboratory, Brazilian Center for Research in Energy and Materials, Giuseppe Maximo Scolfaro, 10000, Campinas, SP 13083-970, Brazil

**Keywords:** *Serratia marcescens*, PgaB, *Staphylococcus aureus*, biofilm degradation

## Abstract

Antimicrobial resistance (AMR) is a critical global health threat, with projections estimating up to 10 million deaths annually by 2050. One of the strategies for developing bacterial AMR is the formation of microbial biofilms (BFs). Thus, enzymes capable of degrading BF exopolysaccharides represent potential tools for BF disruption. In this work, we characterize β-1,6-*N*-acetylglucosaminidase from *Serratia marcescens* (*Sm*PgaB), a two-domain enzyme with covalently attached GH153 and CE4 modules. Small-angle scattering data demonstrate that *Sm*PgaB is monomeric in solution. We also demonstrate that *Sm*PgaB degrades *Staphylococcus aureus* biofilms with up to 92% efficiency and inhibits biofilm formation by over 95%. Furthermore, *Sm*PgaB enhances the effectiveness of gentamicin, tetracycline, and chloramphenicol, reducing the viability of planktonic cells by approximately 50% when used in combination with these antibiotics. Confocal laser scanning microscopy confirmed considerable morphological changes in the biofilm post-treatment. These results showcase the potential of β-1,6-*N*-acetylglucosaminidases as adjunct therapies for BF-related infections, particularly when combined with conventional antibiotics.

## Graphical abstract

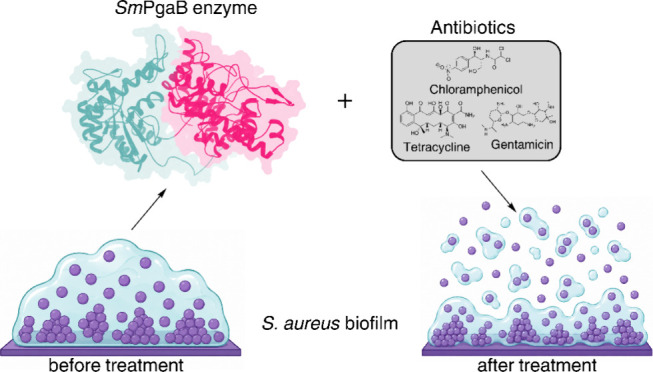

## Introduction

Antimicrobial resistance (AMR) has become one of the most pressing global health challenges. Among major contributors to AMR are bacterial biofilms (BFs), formed when communities of microorganisms attach to inert or living substrates by an extracellular matrix, primarily composed of self-produced exopolysaccharides (EPS), extracellular DNA (eDNA), and proteins.^1^ The extracellular matrix reduces antibiotic penetration, enhances tolerance to stress, and protects embedded cells from immune clearance, rendering biofilm-associated infections markedly more difficult to eradicate than planktonic cells.^2^

*Staphylococcus aureus* is a Gram-positive, BF-forming pathogenic bacterium commonly found in the upper respiratory tract of humans. This opportunistic pathogen is characterized by its ability to form a BF, which, once established, becomes recalcitrant to antibiotic treatments, making it a recurrent agent of infections.^3^ A key component of *S. aureus* biofilm composition is partially de-*N*-acetylated poly-β-1,6-*N*-acetyl-d-glucosamine (PNAG), also known as polysaccharide intercellular adhesin (PIA).^4^ PNAG is synthesized by the enzymes encoded by the icaADBC locus^5^ in Gram-positive bacteria, whereas in Gram-negative bacteria, it is produced by the enzymes encoded by the pgaABCD operon,^6^ and is conserved across several pathogens, including **Escherichia coli*, *Acinetobacter baumannii**, and *Staphylococcus epidermidis*.^7^ Its widespread distribution and essential roles in adherence to biotic and abiotic surfaces, bacterial intercellular adhesion, and evasion of host immune defenses^8^ make PNAG a central target for biofilm-disrupting strategies.

Since PNAG forms the dominant structural scaffold of many BFs, the enzymes capable of modifying or degrading this polymer have emerged as promising therapeutic tools.^9,10^ Several carbohydrate-active enzymes (CAZymes) have been explored to degrade EPS matrices and potentiate antibiotic activity, including DNase I for eDNA disruption, proteinases for matrix-protein degradation, and glycosidic enzymes such as dispersin B and its homologues^11,12^ and PgaBs,^13–17^ which cleave PNAG. The structure of these enzymes consists of two distinct domains: an N-terminal domain with deacetylase activity, classified under carbohydrate esterase family 4 (CE4), and a C-terminal domain, a member of the GH153 family, involved in PNAG hydrolysis.^13–17^ Thus, PgaBs potentially offer distinct advantages in PNAG-degradation, as they are capable of specifically and simultaneously altering the degree of PNAG acetylation and hydrolyzing its β-1,6-glycosidic linkages. Despite comprising over 4000 members, this relatively new enzyme family remains very poorly studied. To date, only the enzymes from *Bordetella bronchiseptica* (*Bb*PgaB),^16^ *Escherichia coli* (*Ec*PgaB),^13^ and *Klebsiella aerogenes* PgaB (*Ka*PgaB)^17^ have been biochemically and structurally analyzed. This creates a substantial gap in understanding of PgaB structural diversity, substrate specificity, and efficiency of PNAG-dependent microbial biofilm degradation, which limits their translational development as antibiofilm agents.

To address this challenge, the present study focuses on PgaB from *Serratia marcescens* (*Sm*PgaB), a Gram-negative bacterium that produces PNAG-rich biofilms and is phylogenetically significantly divergent from the previously studied PgaB members. Characterization of biochemical and structural features of *Sm*PgaB, as well as its degradation capacity of PNAG-rich *S. aureus* biofilms, provides an opportunity to expand the comprehension of the molecular bases of PgaB EPS hydrolysis beyond the three previously studied PgaB enzymes. Accordingly, the objective of this work was to clone, express, biochemically, and structurally characterize *Sm*PgaB and to assess its PNAG-degrading activity in the context of *S. aureus* biofilms. These studies aim to advance the understanding of PgaB enzyme diversity and to evaluate their potential as enzymatic tools for PNAG-targeted biofilm degradation.

## Results and Discussion

### *S. marcescens* Sequence Analysis, Cloning, Expression, and Purification

Phylogenetic analyses of GH153 and CE4 proteins identified six major clusters (Supporting Information, Figures S1–S3), with *S. marcescens* located in the largest cluster. Other clusters comprising more specialized or divergent groups showed lower sequence similarity to *S. marcescens*. Analysis of characterized GH153 and CE4 sequences from CAZy revealed three major clusters, with *S. marcescens* clustered with bacterial enzymes from *Aeromonas, Staphylococcus, Ammonifex, Bordetella*, and *Escherichia*. Fungal and aquatic bacterial sequences formed distinct, more-divergent clades.

The *Sm*PgaB gene (GenBank ID: QTI64407.1; UniProt Identifier: A0A2 V4FWX5) was successfully cloned into the pETTRXA-1a/LIC expression vector and expressed in the *E. coli* BL21 strain. The enzyme purification yielded around 50 mg per liter of culture medium, with a high level of purity ascertained by 12% SDS-PAGE analysis (Supporting Information, Figure S4). The nonfused enzyme migrated as a single band with an approximate molecular mass of 72 kDa, which is close to the theoretical molecular mass of 73.292 kDa predicted from its amino acid sequence.

### Biochemical Characterization of *Sm*PgaB

The optimal temperature and pH for *Sm*PgaB were measured using the BF from the *S. aureus* 3 strain. Although the maximum activity was observed at pH 5.0, the error bars indicate that the enzyme exhibits essentially equivalent activity across a broad plateau between pH 4.0 and 7.0 (Figure 1a). The enzyme maintained more than 95% of its maximum activity in the temperature range of 20–40 °C. A clear decline in *Sm*PgaB enzymatic activity is observed at temperatures above 60 °C (Figure 1b). To evaluate residual enzymatic activity under both application-relevant and stress conditions, its activity was subsequently tested as a function of time at 40 °C (proximal to host body temperature) and at 70 °C (where activity markedly decreases). At 40 °C, the enzyme was highly stable, retaining more than 90% and 80% of its activity after 3 and 24 h of incubation, respectively (Figure 1c). At 70 °C, the enzyme lost 60% of its enzymatic activity after 15 min of incubation and became completely inactive after 3 h (Figure 1c).

**Figure 1:**
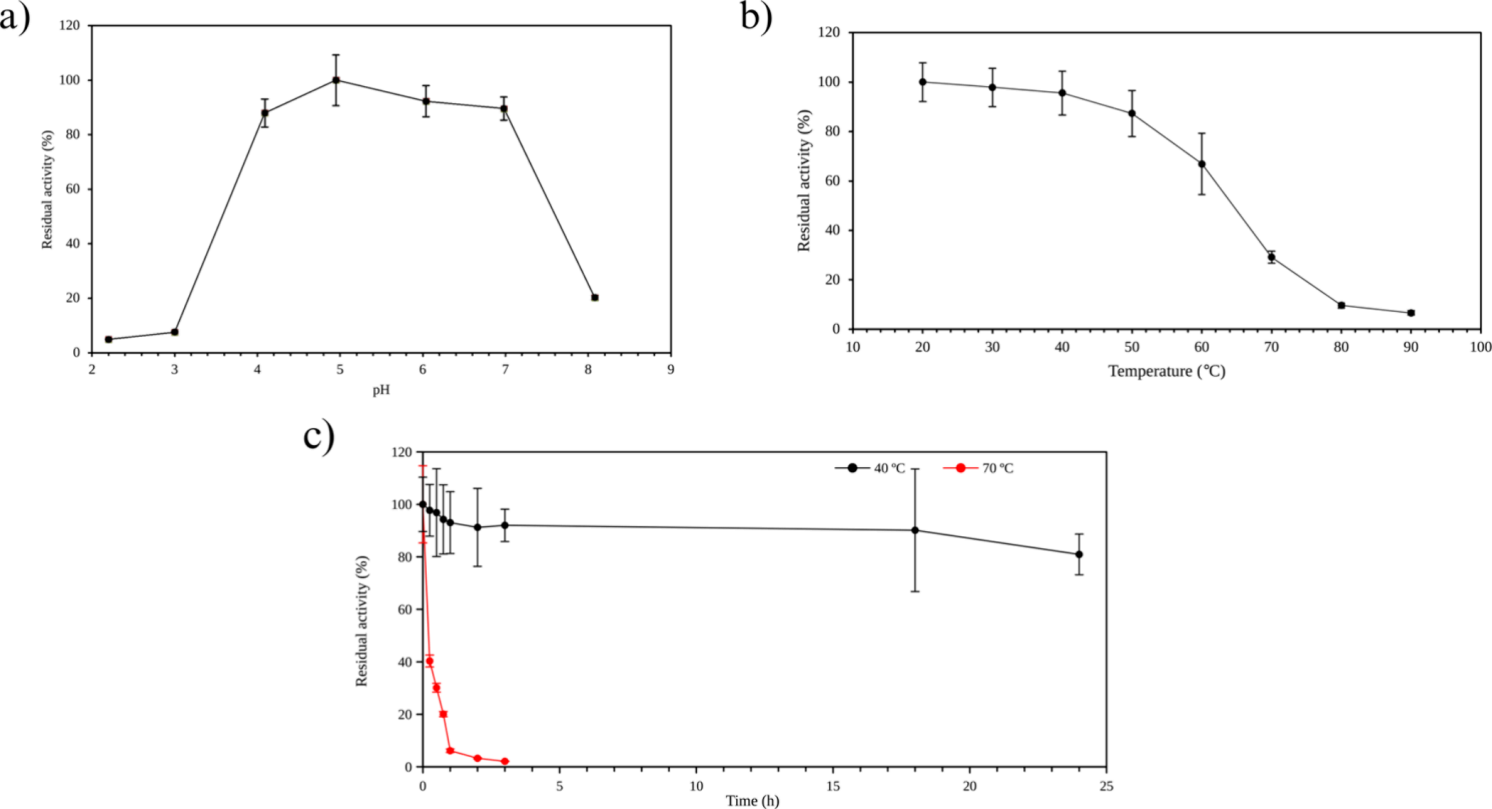
Optimal conditions as a function of pH, temperature, and incubation time. (a) pH profile; (b) residual activity in relation to temperature; (c) residual activity after different periods of incubation at 40 and 70 °C. Error bars represent standard deviations (SD) from six replicates.

Thus, the optimal characteristics of *Sm*PgaB are consistent with its bacterial origin. *S. marcescens* is a mesophilic bacterium with optimal growth temperatures ranging from 30 to 40 °C and pH from 6.5 to 8.0. Residual activity results are also consistent with ThermoFluor assays and circular dichroism analysis of the enzyme (Supplementary Figure S5).

### Analysis of Structural Stability Using Thermal Shift Assay

*Sm*PgaB was subjected to a thermal shift (ThermoFluor) assay using 48 different buffers. This assay demonstrated that the highest melting temperature was 70 °C when a potassium phosphate buffer at pH 7.0 was used, and the enzyme maintained an average melting temperature of 55 °C in the pH range 6.0–8.0 in the absence of salt (Supplementary Figure S5a). However, when the buffers contained salt, the melting temperature in the same pH range decreased by 3 °C (Supplementary Figure S5a). Furthermore, in the presence of NaCl, the average *T*_m_ decreased by 1 °C. *Sm*PgaB was negatively affected by the presence of salt, suggesting that its structural stability might be negatively impacted by high ionic strength environments.

### Structural and Binding Sites Analysis

*Sm*PgaB 3D structure was successfully predicted by AlphaFold.^18^ The predicted model of *Sm*PgaB was compared with the crystallographic model of the two homologous enzymes with known 3D structure, *Ec*PgaB from *Escherichia coli* (PDB ID: 4P7R)^14^ and *Bb*PgaB from *Bordetella bronchiseptica* (PDB ID: 6AU1).^15^ The sequence identity between *Sm*PgaB and these two enzymes is 52.88% and 45.14%, respectively. In addition, the root-mean-square deviations (RMSDs) between *Sm*PgaB and the X-ray structures of *Escherichia coli* and *Bordetella bronchiseptica* are 0.61 and 0.81 Å, respectively.

The *Sm*PgaB structure has two distinct structural domains (Figure 2a): an N-terminal carbohydrate esterase CE4 domain and a C-terminal glycoside hydrolase GH153 domain connected by a short CE4/GH153 interdomain linker.

**Figure 2:**
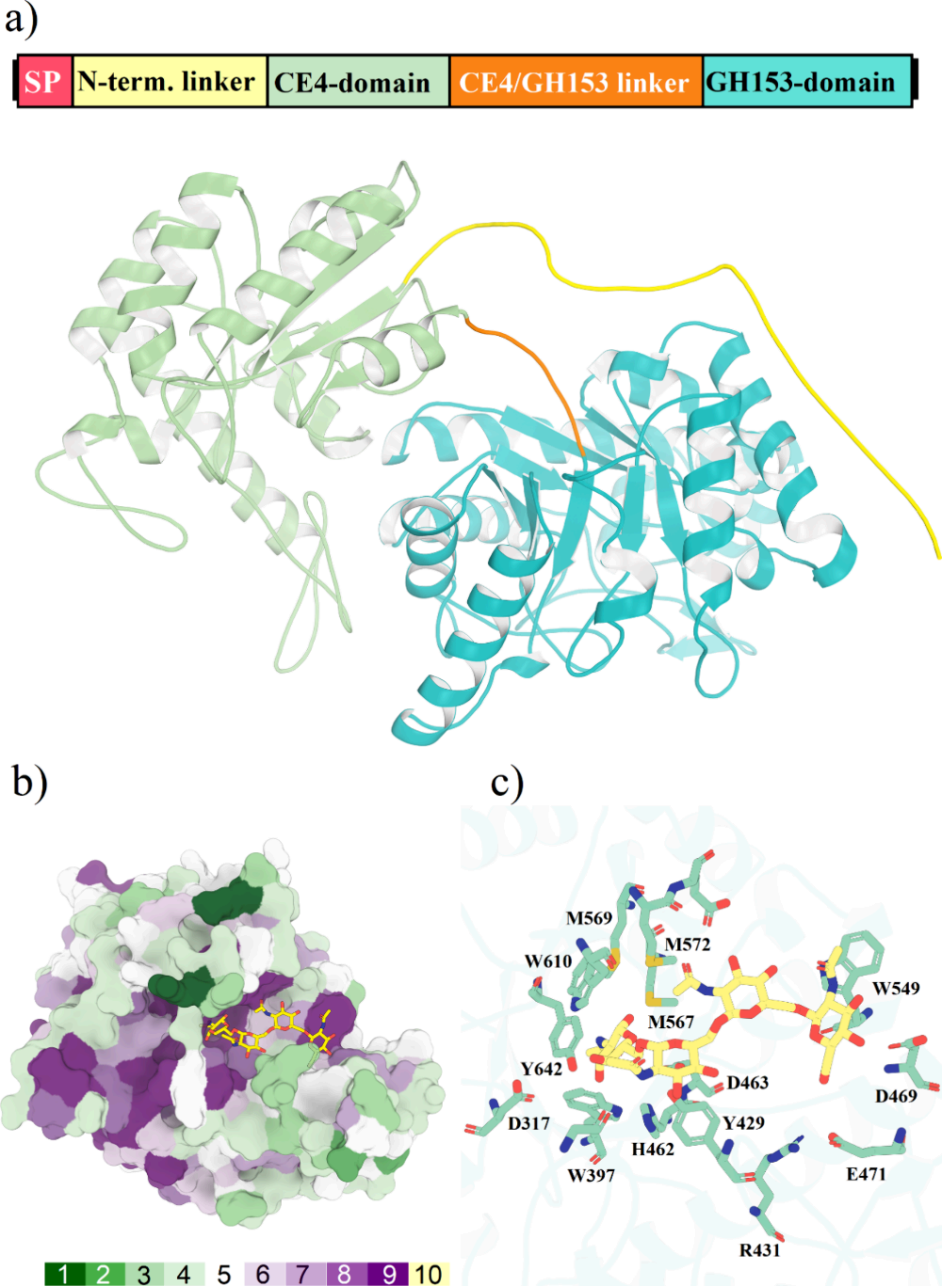
Structural analysis of *Sm*PgaB. (a) Domain architecture and AlphaFold-predicted structure of full-length *Sm*PgaB. The linear schematic (top) highlights the multidomain organization: signal peptide (SP, red), N-terminal linker (yellow), CE4 domain (green), CE4/GH153 linker (orange), and GH153 domain (cyan). The 3D structural model (bottom) reveals independently folded CE4 and GH153 catalytic domains connected by a flexible linker; (b) molecular surface representation of the GH153 domain with docked PNAG oligomer (yellow sticks). Residues are color-coded based on evolutionary conservation scores from ConSurf analysis,^21^ ranging from variable (green) to highly conserved (purple), with the conservation scale shown below. The PNAG-binding groove is enriched with highly conserved residues; (c) ConSurf conservation mapping across the GH153 domain shows that the PNAG-binding cleft is composed largely of residues with the highest conservation scores (9–10). Close-up of the GH153 active site with conserved residues identified by ConSurf analysis labeled and visualized in stick representation, showing conserved key residues that may participate in catalysis or substrate stabilization.

The both CE4 and GH153 domains form independently folded globular structures, connected via a flexible, nonstructured short linker. The CE4 domain is homologous to *Ec*PgaB N-terminal and *Bb*PgaB N-terminal domains and adopts a circularly permuted arrangement of the CE4 motifs, presenting 7-fold α/β-barrel with three β-hairpin motifs, six α-helices, with one α-helix capping the bottom of the (α/β)_7_ barrel.^14,19^

The C-terminal domain of *Sm*PgaB has an (α/β)_8_ TIM barrel fold.^20^ It exhibits a well-defined catalytic groove cavity that likely serves as the active site for PNAG hydrolysis. This cavity can fit the PNAG polymer in an extended conformation. The groove itself is semienclosed, forming a channel-like structure that may accommodate several sugar units at once, enabling the hydrolysis of deacetylated PNAG.

To investigate substrate recognition, molecular docking modeling of poly-β-1,6-*N*-acetylglucosamine (PNAG) into the GH153 domain was performed (Figure 2b,c). The most favorable docking pose for a PNAG hexamer yielded an estimated binding energy (Δ*G*) of −6.92 kcal/mol. The docked PNAG ligand (yellow sticks) is localized within a surface-accessible catalytic cleft pocket of the GH153 domain. The docking analysis shows that PNAG aligns along a trench enriched in highly conserved residues, indicating the functional importance of this surface for substrate engagement. This spatial correlation between ligand binding and evolutionary conservation supports the role of this active site for GH153 enzymes that process PNAG polysaccharide substrates via glycosidic bond cleavage. The geometry of the GH153 groove is particularly suited for binding linear β-1,6-linked PNAG. Aromatic residues, including W397, W549, W610, and Y642, are arranged to permit face-to-face and edge-to-face interactions with the sugar rings of PNAG within the catalytic cleft. Acidic residues such as D463, D469, and E471 lie at the base of the groove and are well-positioned to act as catalytic residues. Based on their proximity to the scissile glycosidic bond in the docked model, D463 and E471 may function as a general acid and base pair, respectively, to facilitate proton transfer and nucleophilic attack during bond cleavage. H462 may also potentially stabilize the substrate via interaction with the substrate hydroxyl groups. Indeed, the conserved and predicted catalytic residue D463 is located in this region, which is equivalent to D466 in *Ec*PgaB and D474 in *Bb*PgaB. The conservation and clustering of these residues across homologues strongly support their catalytic relevance.

To further validate the functional role of this groove, the *Sm*PgaB GH153 domain was structurally aligned with crystallographic structures of homologous glycoside hydrolases in the Protein Data Bank that contain *N*-acetylglucosamine (NAG) ligands in their active sites. The alignment revealed consistent positioning of multiple NAG ligands within the same cleft as the docked PNAG (Figure 3a). These NAG molecules, derived from experimentally determined X-ray structures, align precisely along the conserved groove of the *Sm*PgaB GH153 domain, reinforcing their role as the substrate-binding tunnel. Importantly, the ligands occupy both shallow and deep positions along the groove, mimicking a potential stepwise degradation pathway for the PNAG chains.

**Figure 3:**
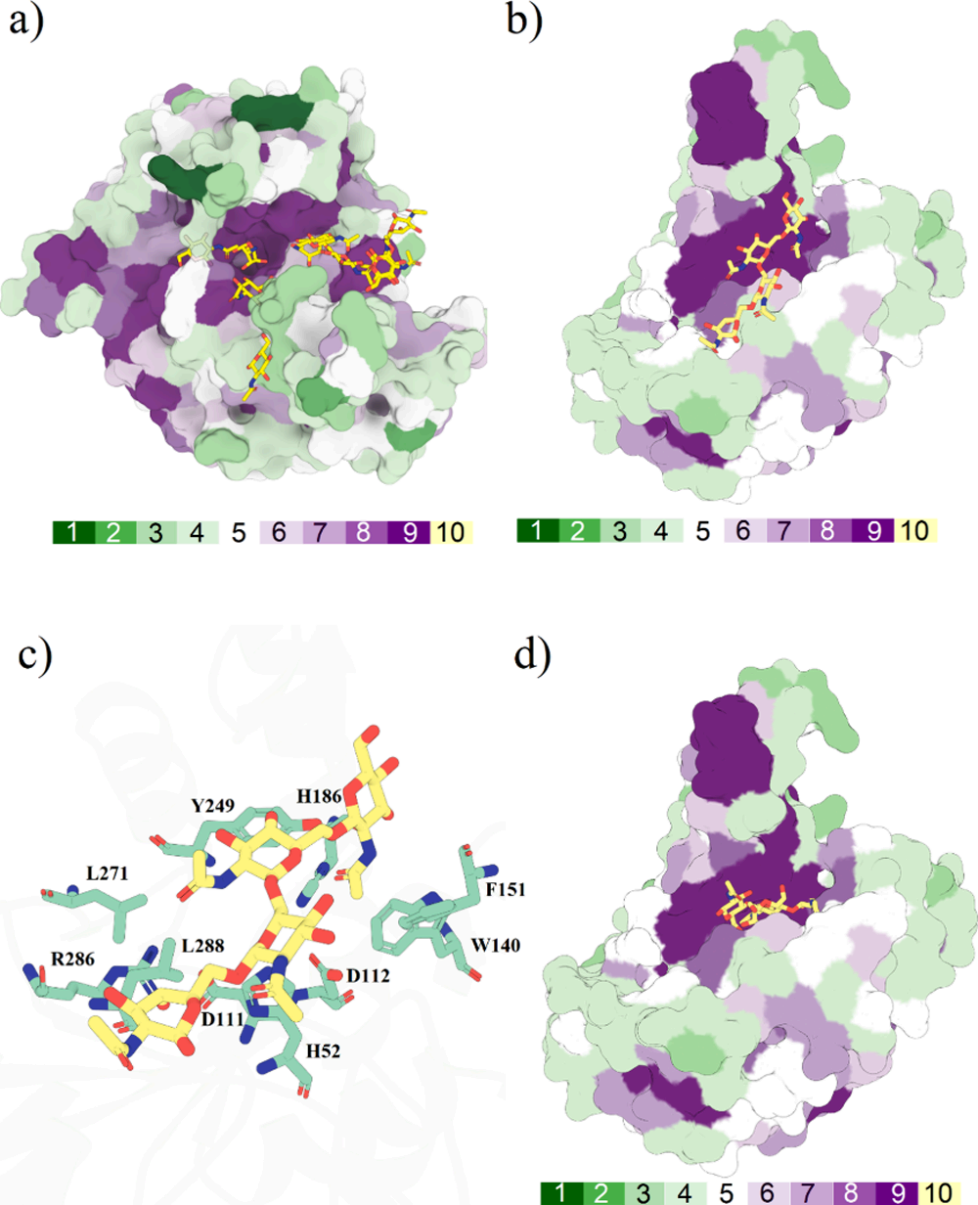
Structural analysis of *Sm*PgaB. (a) Ligand binding sites obtained from the structural alignment of the GH153 domain with homologous glycoside hydrolases (PDB ids: 4P7Q and 4P7R). Multiple ligands (yellow sticks) map onto the same conserved groove observed in the GH153 domain of *Sm*PgaB model, validating its role as the active site for glycosidic bond cleavage. (b) Surface rendering of the CE4 domain with a docked PNAG fragment (yellow sticks) positioned within a deep groove. Surface coloring reflects evolutionary conservation scores calculated via ConSurf,^21^ where highly conserved residues (score 9) cluster around the binding groove. (c) Close-up of the CE4 active site, highlighting conserved catalytic and substrate-binding. (d) Structural alignment of the CE4 domain with homologous CE4 enzymes containing bound *N*-acetylglucosamine ligands. Experimental CE4 structures with ligands align within the same conserved groove, supporting structural conservation of the deacetylase active site across the CE4 family. Conservation scores calculated using ConSurf.^21^ Ligands in (d) were obtained by structural alignment with CE4 homologues from the Protein Data Bank (PDB) with bound GlcNAc derivatives (PDB ids: 4OUI and 4NZ1) with the CE4 domain of *Sm*PgaB.

In addition, the *Sm*PgaB GH153 domain features eight small α-helices on its top face that generate an electronegative surface formed by residues D317, D319, Y320, Q351, D355, K357, D359, W397, K427, Q428, H462, D463, D464, L466, D469, Y537, W549, F550, M567 and Y642, which encapsulate a groove measuring 39.5 Å in length and 15 Å in width. The groove also envelops the central pocket, a region measuring 20.7 Å in depth and 9.6 Å in width. As expected, the residues in this region are highly conserved (scores 9–10 obtained with ConSurf analysis^21^) among previously characterized orthologous enzymes, consistent with its role in substrate binding (Supplementary Figure S6).

Similar to *Bb*PgaB,^15^ *Sm*PgaB features a more open active site when compared to *Ec*PgaB. Loop 7 in the *E. coli* enzyme almost completely occludes the active site, while *Sm*PgaB has a more open structure resembling that of *B. bronchiseptica* enzyme, which could facilitate the binding of complex and decorated substrates.^15^

Structural analysis of the CE4 domain of *Sm*PgaB with PNAG docked into its active site reveals specific residues predicted to interact directly with PNAG (Figure 3b). Surface representation and conservation analysis (Figure 3b–d and Supplementary Figure S6) reveal a prominent, conserved groove that runs along one face of the domain. Docking of a PNAG fragment modeled into this groove shows that the substrate aligns well within a trench of strongly conserved residues, indicating this site serves as the deacetylase active site. Conservation mapping analysis shows that the majority of residues within this groove have high conservation scores (Figure 3c), reinforcing their functional importance in substrate recognition. Detailed visualization of the active site identifies key catalytic and binding residues arranged around the docked substrate. H52, D111, and D112 form the canonical His-Asp-Asp triad, typical of CE4 family deacetylases, and are well-positioned to coordinate a catalytic water molecule for nucleophilic attack on the *N*-acetyl group of GlcNAc residues as observed in the crystal structure of *Ec*PgaB (PDB ID: 4P7R)^14^ and *Bb*PgaB (PDB ID: 6AU1).^15^ The active site of CE4 family members is located at the center of the shallow barrel-like β-sheet (Figure 3d), with the predicted active-site residues D112 and H181 being equivalent to residues D115 and H184 from *Ec*PgaB and *Bb*PgaB.^19^ These residues are also involved in the coordination of Co^2+^and Ni^2+^ ions.^15^

Moreover, the structural alignment of the *Sm*PgaB CE4 domain with homologous CE4 domain-containing enzymes reveals a conserved ligand binding architecture (Figure 3d). Acetylated GlcNAc ligands from these experimentally solved structures superimpose closely with the docked PNAG fragment, occupying the same catalytically active site. This overlap between the modeled and experimental ligand positions confirms the structural and functional conservation of this active site across the CE4 family. These observations suggest that the CE4 domain of *Sm*PgaB acts as a PNAG-specific deacetylase, removing *N*-acetyl groups from GlcNAc residues to generate partially deacetylated PNAG, which is required for the downstream hydrolysis by the GH153 domain.

The linker between the N-terminal and C-terminal domains is only ∼7 residues, similar to that of *Ec*PgaB. This short linker may indicate a more rigid interdomain association compared to *Bb*PgaB, which has a linker approximately twice as long.^15^

### SEC-SAXS Experiments and Structural Analysis

To assess the solution structure of *Sm*PgaB, we performed in-line size-exclusion chromatography (SEC) combined with small-angle X-ray scattering (SAXS) to determine the molecular shape and the conformational properties of the enzyme. The experimental SAXS-derived parameters are given in Supplementary Table S1. In the Guinier approximation, the scattering curve of the enzyme exhibited a linear behavior in the initial *q*-region (Figure 4a), confirming the absence of aggregation in the analyzed data. The radius of gyration (*R*_g_) values obtained from the Guinier analysis were 28.56 ± 0.07 Å.

**Figure 4:**
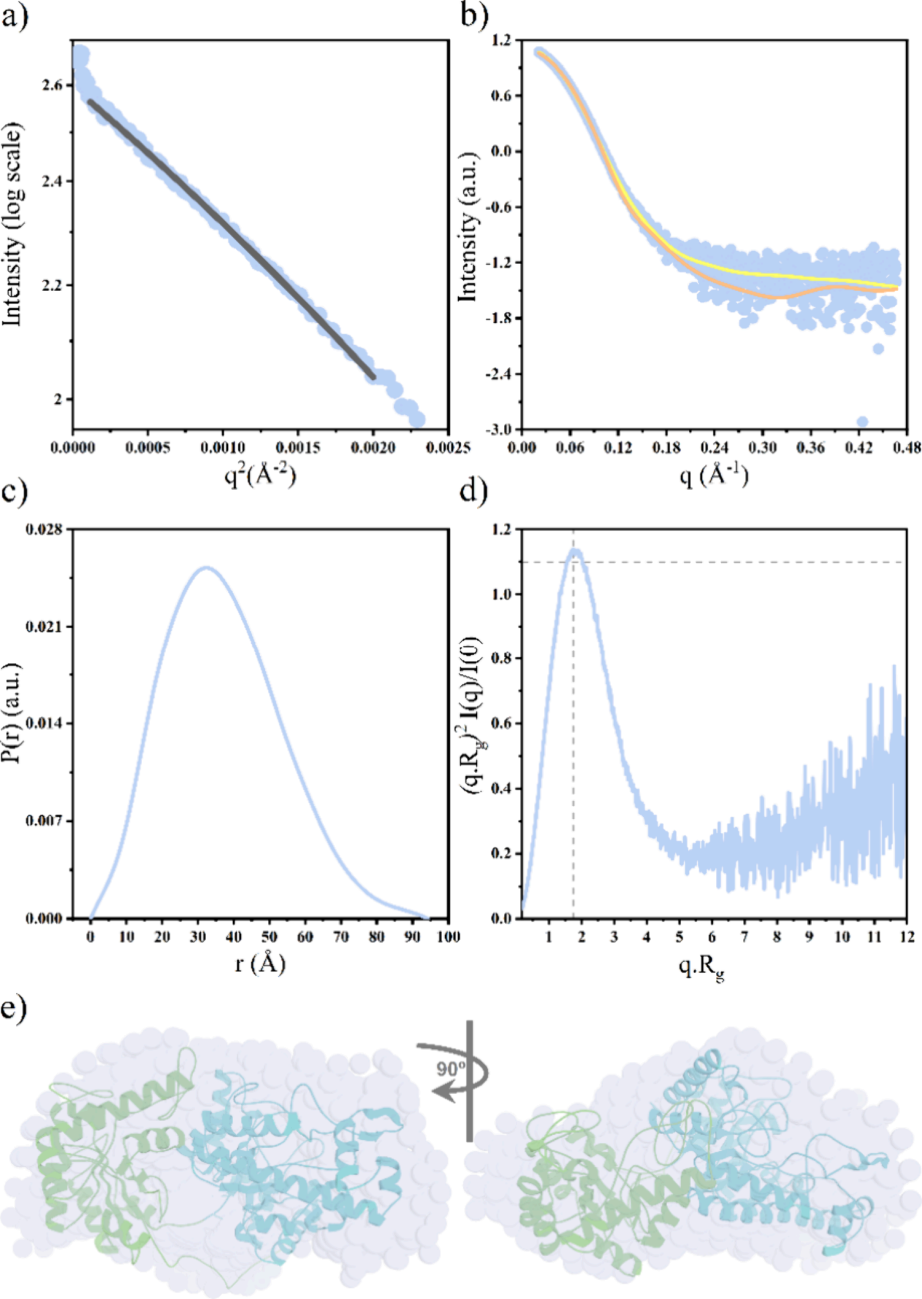
SAXS data processing and analysis of *Sm*PgaB. (a) Guinier plot showing the linear region used to estimate *R*_g_ for *Sm*PgaB; (b) Comparison of experimental SAXS intensity (blue circles) with theoretical scattering curves obtained from DAMMIN (yellow) and AF3 (orange). (c) *P*(*r*) function determined from the experimental scattering data for *Sm*PgaB. d) Kratky plot, indicating the degree of structural flexibility of *Sm*PgaB. Dashed lines were drawn at *q*. *R*_*g*_ = 1.73 and (*q*. *R*_*g*_)^2^ *I*(*q*)/*I*(0) = 1.104; (e) low-resolution model of *Sm*PgaB (gray spheres). The SAXS-derived molecular envelopes were obtained via DAMMIN, and they were superimposed with their respective AlphaFold models, color-coded by domains with green (CE4) to teal (GH153).

The experimental scattering intensity (Figure 4b) was fitted against theoretical curves generated from different structural models, including DAM (*ab initio* reconstruction from SAXS experimental intensity) and AlphaFold-derived models (AF3).^22^ A χ^2^ analysis indicated a better fit to the data for the DAM models (χ^2^ = 1.92) compared to the AlphaFold-based model (χ^2^ = 5.47). Additionally, the molecular weight estimated from the scattering intensity using SAXSMoW 2.0 ^23^ was 69.8 kDa, representing a discrepancy of only 4.7% from its theoretical sequence-based molecular mass. In addition, size-exclusion chromatography (SEC) with multi-angle light scattering (MALS) measurements confirmed that *Sm*PgaB is a stable monomer with a molecular weight of 71.7 kDa in solution (Supplementary Figure S8).

The pair distribution function P­(r) (Figure 4c) exhibits a unimodal *P*(*r*) distribution with a maximum particle dimension (*D*_max_) of 94.34 Å. The real-space *R*_g_ calculated from *P*(*r*) was 28.68 ± 0.04 Å, closely matching the value obtained from the Guinier approximation.

The normalized (dimensionless) Kratky plot analysis (Figure 4d), shown as (*q*. *R*_*g*_)^2^ *I*(*q*)/*I*(0) versus *q*. *R*_*g*_, provides insight into the global conformation and flexibility of *Sm*PgaB in solution. Globular proteins are expected to exhibit a local maximum at position *q*. *R*_*g*_ = 1.73 and height of (*q*. *R*_*g*_)^2^ *I*(*q*)/*I*(0) = 1.104 with a bell-shaped peak,^24^ and deviations from the ideal bell-shaped intensity profile indicate extended conformation or protein flexibility or disorder.^25,26^ *Sm*PgaB exhibited a Gaussian-like peak at low *q*, with a maximum at (*q*. *R*_*g*_)^2^ *I*(*q*)/*I*(0) = 1.13 and *qR*_*g*_ ≈ 1.7 3 that converges toward higher scattering vector values (1 ≤ *q*. *R*_*g*_ ≤ 12), which is characteristic for compact and well-folded proteins. However, the curve profile does not decay to zero at higher *q*. *R*_*g*_ values (6–7 ≤ *q*. *R*_*g*_ ≤ 12). It displays a minimum followed by an elevated baseline at *q*. *R*_*g*_ ≈ 6–7, consistent with some flexibility introduced by interdomain motions.

The *ab initio* modeling was performed using the software DAMMIF^27^ and DAMMIN.^28,29^ Each generated model was evaluated against the experimental data by comparing their predicted scattering curves. The SAXS envelope of *Sm*PgaB (Figure 4e) show excellent superposition with the AlphaFold model, with χ^2^ of 1.92. The SAXS-derived molecular envelope indicates that *Sm*PgaB adopts an elongated architecture in solution (Figure 4e). The envelope is characterized by two well-defined lobes connected by a narrow linker region, consistent with the enzyme two-domain organization. The AF3-derived atomic model (Supplementary Figure S9a) is fully accommodated within the SAXS-derived bead model, indicating that the high-resolution structure accurately represents the protein conformation in solution. The relative orientation of the two domains appears constrained, suggesting limited interdomain flexibility as observed in the Kratky plot (Figure 4d).

To investigate the interdomain mobility of *Sm*PgaB in solution, the flexibility of the enzyme was studied by using comparative conformational analysis using experimental SAXS data and AF3-derived model. Normal mode analysis (NMA) (χ^2^ versus RMSD) was performed in SREFLEX,^30^ while root-mean-square fluctuation (RMSF) was evaluated through fast simulation-based modeling of protein structure flexibility in CABS-flex.^31^ These analyses shed light on the structural integrity, model accuracy, and dynamic flexibility of the proteins in question, offering insights into the structural properties of the *Sm*PgaB in solution.

Fitting of the experimental SAXS scattering intensity against the theoretical scattering curve derived from the NMA model showed a good overall agreement (Supplementary Figure S9b), with a χ^2^ value of 2.25. Analysis across the ensemble of NMA-generated conformations revealed a broad distribution of χ^2^ values (Supplementary Figure S9c). This dispersion indicates that multiple conformations deviate from the experimental profile, consistent with the enzyme conformational heterogeneity.

The residue-specific RMSD plot (Supplementary Figure S9d) quantifies the deviation of each residue’s position from AlphaFold’s predicted model structure. This was obtained from the NMA computed structure that exhibited the lower χ^2^ against the experimental SAXS intensity. *Sm*PgaB shows consistently higher RMSD values, particularly at the N-terminus (residues 18–100), suggesting significant conformational disorder in this region. Higher RMSD values in this region could be indicative of disorder or flexibility at the N-terminal region, leading to deviation from the average SAXS-derived structure. Moreover, the residue-specific RMSF plot (Supplementary Figure S9e) measures the flexibility of individual residues, with higher RMSF values indicating more flexibility over time. Thus, one can conclude that *Sm*PgaB exhibits higher fluctuations and structural disorder at its C-terminal and N-terminal regions. This is consistent with previous crystallographic studies of EcPgaB,^14,32^ which reveals that both C- and N-terminal parts of the enzyme are disordered and have to be enzymatically cleaved off to allow for successful crystallization. Taking into consideration that PgaB is part of the Pga gene cluster responsible for PNAG synthesis and interacts with other proteins of this cluster, such as PgaA,^33^ one might speculate that this structural disorder of its C- and N-terminal fragments is necessary to form stable multiprotein complexes in solution required for PNAG synthesis and translocation. It was reported that PgaA:PgaB complex formation leads to significantly enhanced activities of both deacetylase (2.7 times) and glycoside hydrolase (13 times) activities of PgaB,^33^ further strengthening the importance of the multienzyme interactions of PgaB in biological settings, in which both C- and N-termini of the enzyme might be involved.

### Genomic Studies of *S. aureus* Isolates Used in This Work

The genomes of *S. aureus* isolates studied in this work had ∼2.6 Mb of total length, which were assembled onto up to 5 contigs, with an N50/90 of ∼2.6 Mb, L50/90 of 1, GC content of ∼32.7%, and similarity with the reference genome of >90% (Table 1). These metrics indicate that the desirable genomes were obtained.

**Table 1:** General Metrics of Genome Assembling Calculated Using QUAST. *: Percentage of Similarity with the Reference Genome

strain	length	number of contigs	N50	N90	L50	L90	GC%	Sim. (%)
S. aureus 1	2,681,998	5	2,674,244	2,674,244	1	1	32.72	90.36
S. aureus 2	2,638,297	4	2,630,565	2,630,565	1	1	32.72	90.82
S. aureus 3	2,626,113	5	2,598,718	2,598,718	1	1	32.67	95.36

The circular visualization of the *S. aureus* 1, 2, and 3 genomes demonstrated a structural organization with a high density of coding regions distributed evenly in positive and negative strands (sense and antisense orientations, respectively). The GC content calculated in 1 Kb sliding windows revealed a globally homogeneous nucleotide composition. However, one can also observe localized fluctuations characterized by abrupt deviations in GC percentage relative to the chromosomal average, indicating *loci* of potential genomic variability (Figure 5).

**Figure 5:**
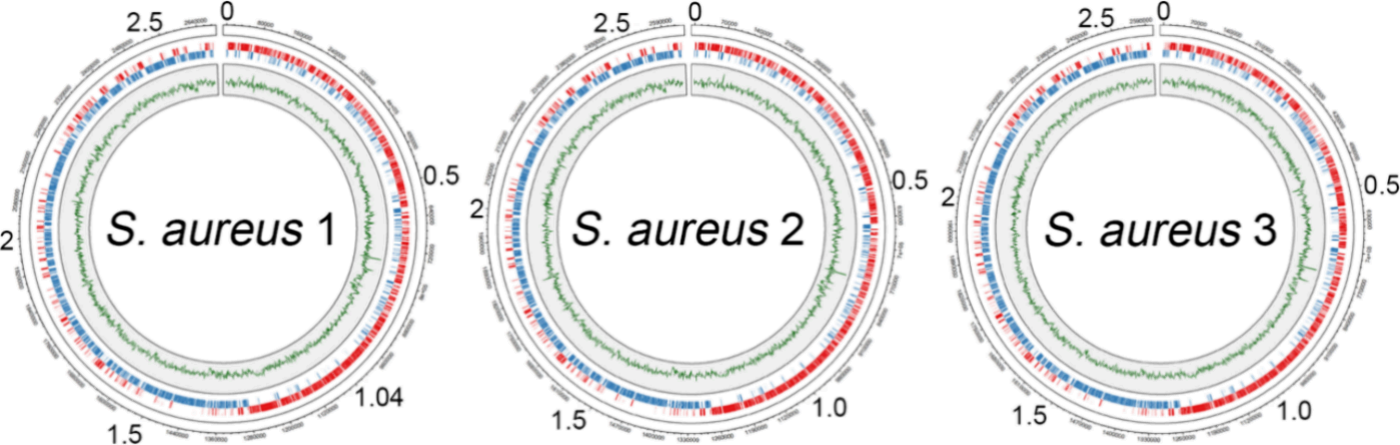
Visualization of *S. aureus* genomes. Red: coding sequences in positive strand; blue: coding sequences in negative strand; green: percentage of GC content.

Synteny analysis based on BLASTn alignments evidenced a high degree of global collinearity among the genomes analyzed, indicating high genomic conservation. The comparison between *S. aureus* 1 and 2 demonstrated a strict preservation of gene order. However, comparison with the *S. aureus* 3 isolate revealed the occurrence of an inversion in regions around ∼0.3–0.4 Mb in length (Figure 6), which does not include the ica operon (this operon in *S. aureus* 3 ranges from 32,046 to 36,184 positions).

**Figure 6:**
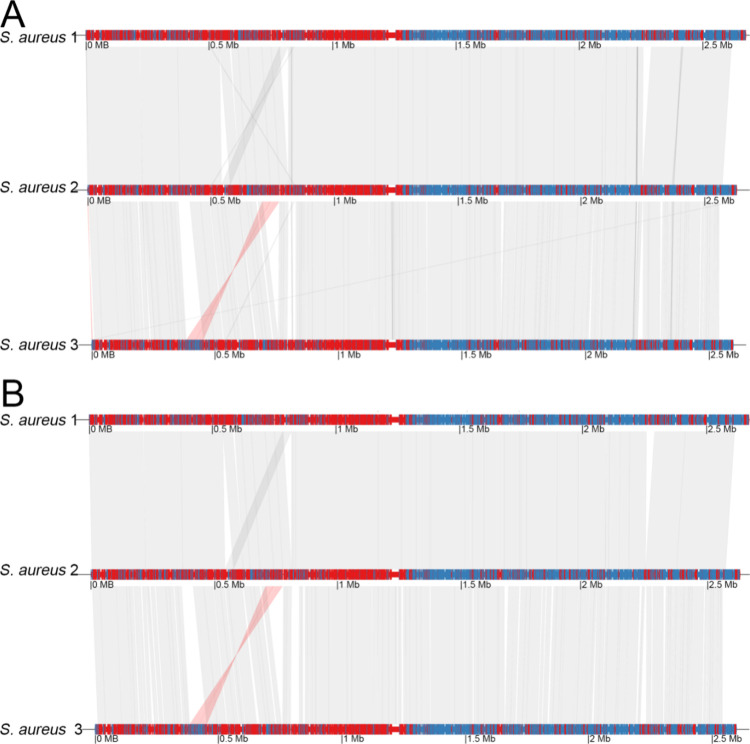
Analysis of synteny. (A) analysis performed excluding artifacts or fragments <2 Kb size; (B) analysis performed excluding artifacts or fragments >5 Kb size; red and blue bars indicate regions with coding sequences in sense and antisense orientation.

Multiple alignment of the regulatory region upstream of the ica operon revealed high nucleotide conservation among isolates, presenting only a single point mutation in *S. aureus* 3 (−144 position) (Figure 7a).

**Figure 7:**
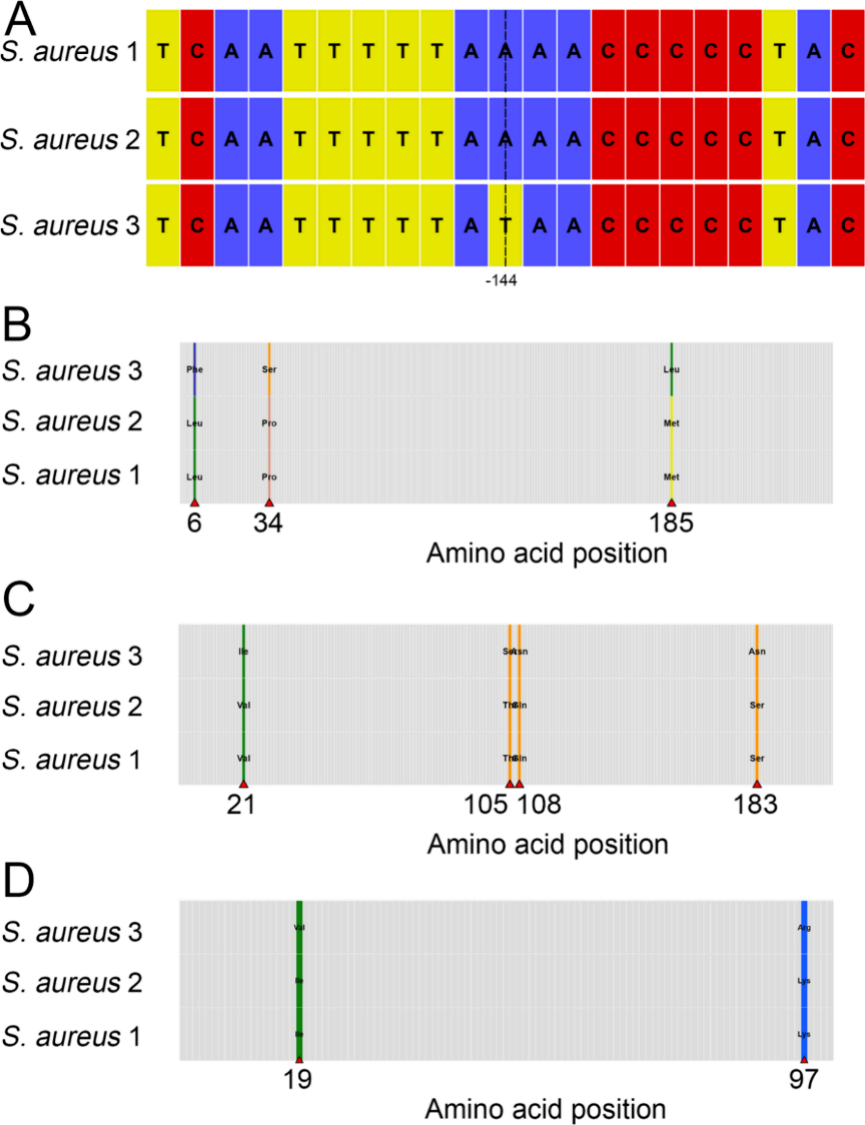
Analysis of the ica operon promoter, and nonsynonymous substitution analysis of ica proteins. (a) Comparison ica operon promoter of analyzed genomes. The number below the alignment depicts the position counted from transcription start site; (b–d) nonsynonymous substitution analysis of icaB, icaC, and ICaD proteins, respectively.

We previously identified that the *S. aureus* 3 genome has a truncated version of the icaR gene (a transcriptional repressor of the ica operon), which is responsible for the unrepression of the ica operon expression culminating in abundant PNAG production.^17^ The IcaA protein presented 100% identity across all analyzed samples, suggesting the maintenance of the functional integrity of this gene. However, IcaB, IcaC, and IcaD proteins of analyzed isolates have up to four nonsynonymous substitutions, including several amino acid substitutions (Figure 7b–d).

### Biofilm Eradication and Inhibition Analysis

To study *Sm*PgaB capacity as an antibiofilm agent, the degradation of three different strains of *S. aureus* by *Sm*PgaB was carried out.

After treatment with the four different doses of the enzyme, a strong decrease in the remaining BF biomass can be observed (Figure 8a–c). The highest BF degradation observed was 40%, 30%, and 92% relative to the growth control for the strains *S. aureus* 1, *S. aureus* 2, and *S. aureus* 3, respectively. The BF of *S. aureus* 3 isolate was most susceptible to *Sm*PgaB enzymatic degradation, consistent with its biofilm matrix rich in PNAG. Contrastingly, strains *S. aureus* 1 and *S. aureus* 2 only demonstrated partial BF eradication, indicating that these strains form biofilms more recalcitrant to PNAG-targeted hydrolysis. This differential susceptibility likely reflects strain-to-strain differences in matrix composition. Although all *S. aureus* strains encode the icaADBC locus responsible for PNAG synthesis, both PNAG abundance and the relative contributions of extracellular DNA and proteins can differ markedly among isolates. The reduced susceptibility of these strains may therefore result from a lower PNAG content, which is consistent with our genomic comparison. Indeed, *S. aureus* 3 isolate contains a premature stop-codon in its Ica repressor, IcaR, which leads to abundant PNAG-rich BF formation. Thus, *S. aureus* forms a BF mostly characterized by PNAG that is composed of partially acetylated β-(1–6) linked glucosamine residues. *S. aureus* 3 produces the densest BF, followed by *S. aureus* 1 and last, *S. aureus* 2 (Figure 8d). To confirm that the *S. aureus* 3 isolate is indeed richer in PNAG as compared to *S. aureus* 1 and 2, we hydrolyzed their BFs with *Klebsiella aerogenes* PgaB enzyme, highly active against PNAG.^17^ The enzymatic hydrolysis indeed was much more efficient against *S. aureus* 3 BF than against *S. aureus* 1 and 2 BFs (Supplementary Figure S10). In addition, we submitted all three BFs to enzymatic hydrolysis with protease (papain) and DNase to reveal the sensibility of the BFs to degradation of their protein and eDNA fractions (Supplementary Figure S10). All three *S. aureus* BFs were significantly degraded by papain. At the same time, DNase had a strong effect on *S. aureus* 1 and 2 BFs, but caused very little impact on *S. aureus* 3 BF. This might indicate that the eDNA component of the latter isolate is strongly protected by the abundant PNAG-based EPS, which renders its DNase hydrolysis ineffective.

**Figure 8:**
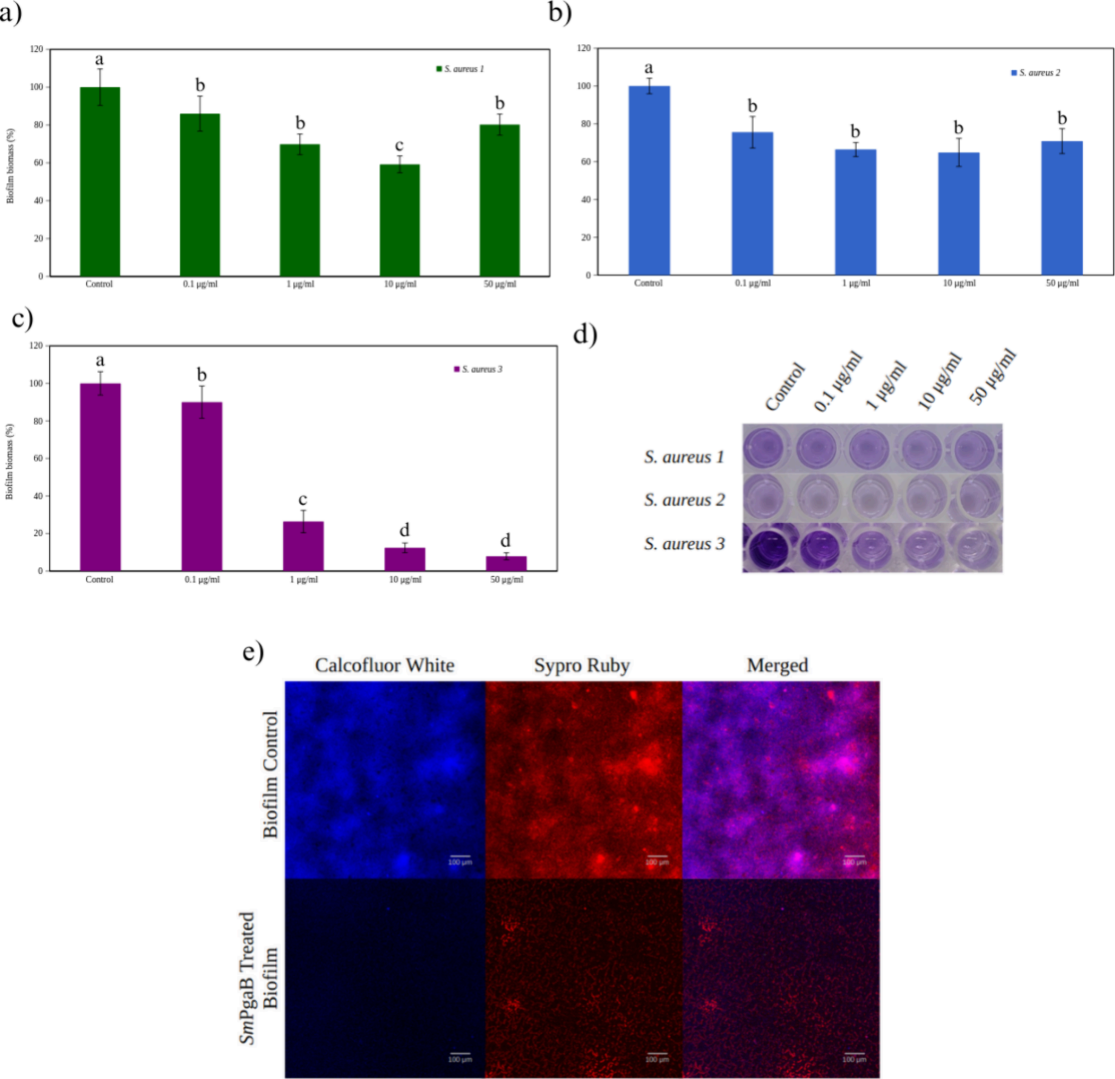
Eradication profile (a) *S. aureus* 1; (b) *S. aureus* 2; (c) *S. aureus* 3; (d) CV staining of studied biofilm biomass after enzymatic treatments; (e) confocal laser scanning microscopy of the *S. aureus* biofilm before and after the treatment with 50 μg/mL of *Sm*PgaB stained with white calcofluor (carbohydrates) and Sypro ruby (proteins). All analyzed biofilms were 24h-old and were analyzed after a 4 h treatment with dosages of 50, 10, 1, or 0.1 μg/mL. All enzymes were dissolved in a 20 mM of phosphate-citrate buffer pH 6 at 37 °C. The results were subjected to a one-way ANOVA statistical tests and Tukey’s HSD post hoc test, which indicate a significant difference between the control and treatments (*p* < 0.01). However, bars with the same letter indicate that *p* > 0.05, showing no significant difference between treatment pairs.

To confirm that the crystal violet assay measured only the residual biomass, the eradicated biofilm was analyzed via confocal laser scanning microscopy (CSLM) to assess any changes in the BF composition after the enzymatic treatment. For such an endeavor, the *S. aureus* 3 biofilm was chosen, as it was the most robust BF studied, which also demonstrated the strongest response to the *Sm*PgaB treatment at a concentration of 50 μg/mL. The control BF is sturdy, with a homogeneous distribution of extracellular matrix and with small clusters of cells (Figure 8e). Additionally, the BF shows a large presence of proteins and PNAG marked by the Sypro Ruby and Calcofluor White stains, respectively. Compared to the treated BF, the blue channel shows a far fainter signal, indicating a strong decrease in carbohydrates after enzymatic treatment. Although *Sm*PgaB does not have any protease-like properties, the diminishment of the signal from the red channel indicates that the proteins in the BF are closely connected with the exopolysaccharides of the extracellular matrix. This observation is also consistent with efficient hydrolysis of *S. aureus* 3 BF by both papain and PgaB.

Next, we followed up with the BF growth inhibition assays. In the BF growth inhibition assays conducted with *S. aureus*, the different strains were exposed to varying doses of *Sm*PgaB, to infer the impact of the dose on the reduction in biofilm formation. The results indicated a significant decrease in biofilm production at the highest concentration for all three strains. *S. aureus* 3 exhibited the largest reduction, showing 95% inhibition of BF formation, while *S. aureus* 1 and *S. aureus* 2 demonstrated moderate reductions of 36% and 27%, respectively (Figure 9). These findings suggest that *Sm*PgaB effectively disrupts biofilm formation across different *S. aureus* strains with varying levels of susceptibility observed among them. Additionally, the results are in accordance with the results found for the eradication assay, which may be indicative of the BF composition, with both *S. aureus* 1 and *S. aureus* 2 presenting less PNAG in their extracellular matrix than the *S. aureus* 3 strain. The data highlight the potential of the enzyme in preventing BF-associated infections, although strain-specific differences in efficacy should be further explored.

**Figure 9:**
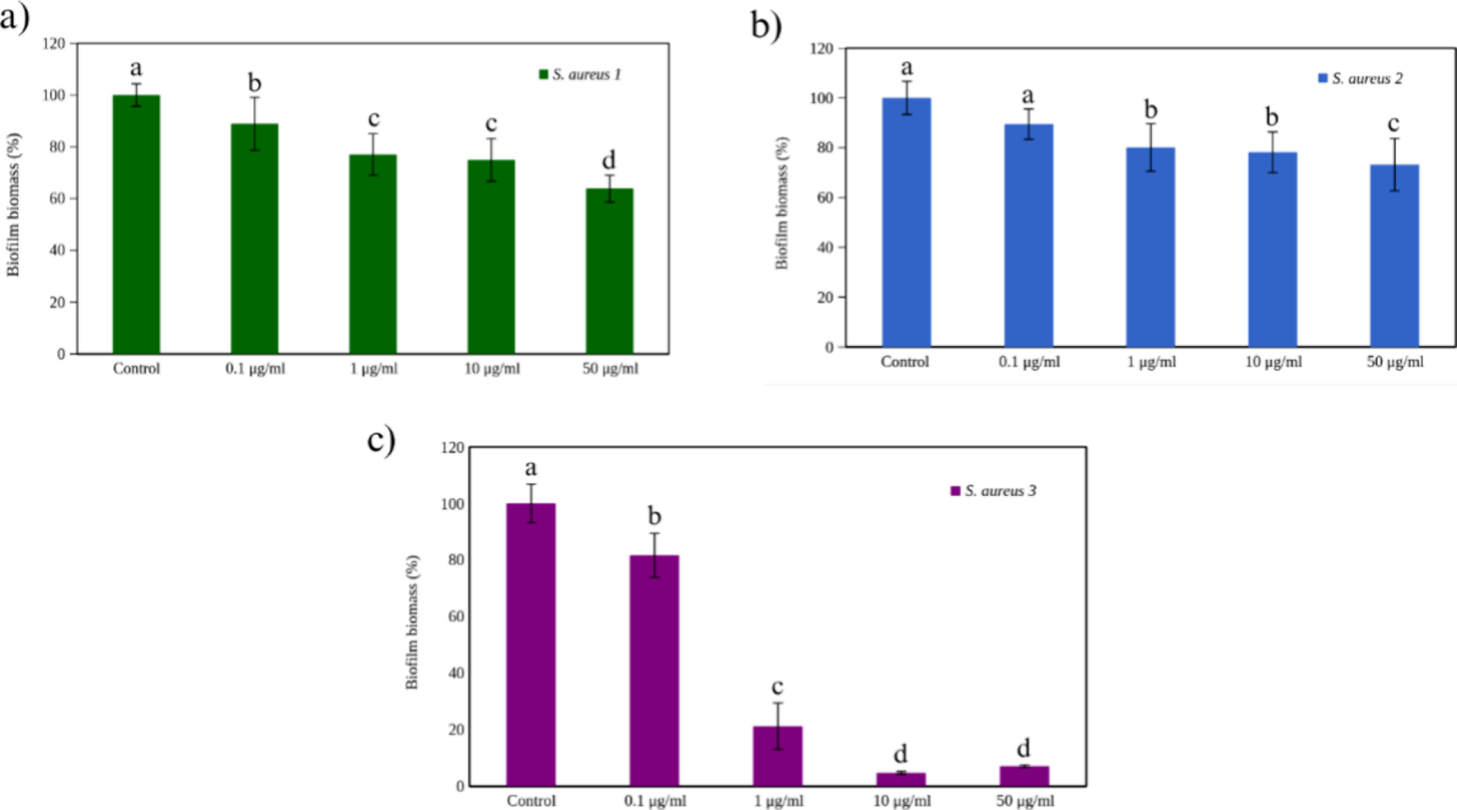
Inhibition profile of (a) *S. aureus* 1; (b) *S. aureus* 2; (c) *S. aureus* 3. All analyzed biofilms were 24 h old and the enzymatic treatment was applied alongside the biofilm inoculum at a dosage of 0.1, 1, 10, and 50 μg/mL. All enzymes were dissolved in an inoculated liquid culture (TSB + 0.75% (w/v) glucose) at 37 °C under static conditions. The results were subjected to a one-way ANOVA and Tukey’s HSD post hoc test, which indicate a significant difference between the control and treatments (*p* < 0.01). Bars with the same letter indicate that *p* > 0.05, showing no significant difference between treatment pairs.

### Changes in Sensitivity to Antibiotics Caused by SmPgaB Biofilm Degradation

In response to the results of the BF degradation by the enzyme, the following study was done using the most robust biofilm-forming strain, *S. aureus* 3. This isolate is resistant to gentamicin, tetracycline, and chloramphenicol according to CLSI/EUCAST breakpoints (gentamicin ≥ 16 μg/mL, tetracycline ≥ 16 μg/mL, and chloramphenicol ≥ 32 μg/mL). Therefore, antibiotics were tested at higher concentrations (50 and 500 μg/mL) to enable detection of effects within this resistant biofilm model. The resazurin cell viability assay examined the effects of each antibiotic alone and combined with *Sm*PgaB to evaluate bacterial survival after a 24 h incubation period. Results showed a significant increase in bacterial cell death when the antibiotics were paired with the enzyme, indicating a synergistic effect, as individually, the antibiotics showed limited activity against the resistant biofilm. Gentamicin, at the concentrations of 50 and 500 μg/mL, showed the most improvement in efficacy with 49% and 55% decrease of viable cells, respectively. Tetracycline showed improvement with 46% and 52%, whereas chloramphenicol effect was improved on 36% and 41% at the concentration of antibiotics of 50 and 500 μg/mL, respectively (Figure 10a,c,e). The concentration of the enzyme was maintained at 50 μg/mL during these experiments. Curiously, for the studied isolates, a 10-fold increase in the dose of analyzed antibiotics (from 50 to 500 μg/mL) did not significantly improve their efficiency alone or when combined with the *Sm*PgaB. This further supports the conclusion that enzymatic biofilm disruption is the dominant factor in these experiments, enabling the antibiotic access. Biofilm-resident cells might display reduced metabolic activity and intrinsic tolerance, which can limit antibiotic efficacy even at high concentrations. Moreover, the *S. aureus* 3 strain is resistant to the tested antibiotics, implying that increases in antibiotic concentrations do not necessarily translate into increased reduction of cell viability. These findings suggest that combining antibiotics with BF-degrading enzymes could be an effective strategy for overcoming the protective barrier of BFs and improving the treatment of biofilm-associated infections caused by *S. aureus*.^16,34^

**Figure 10:**
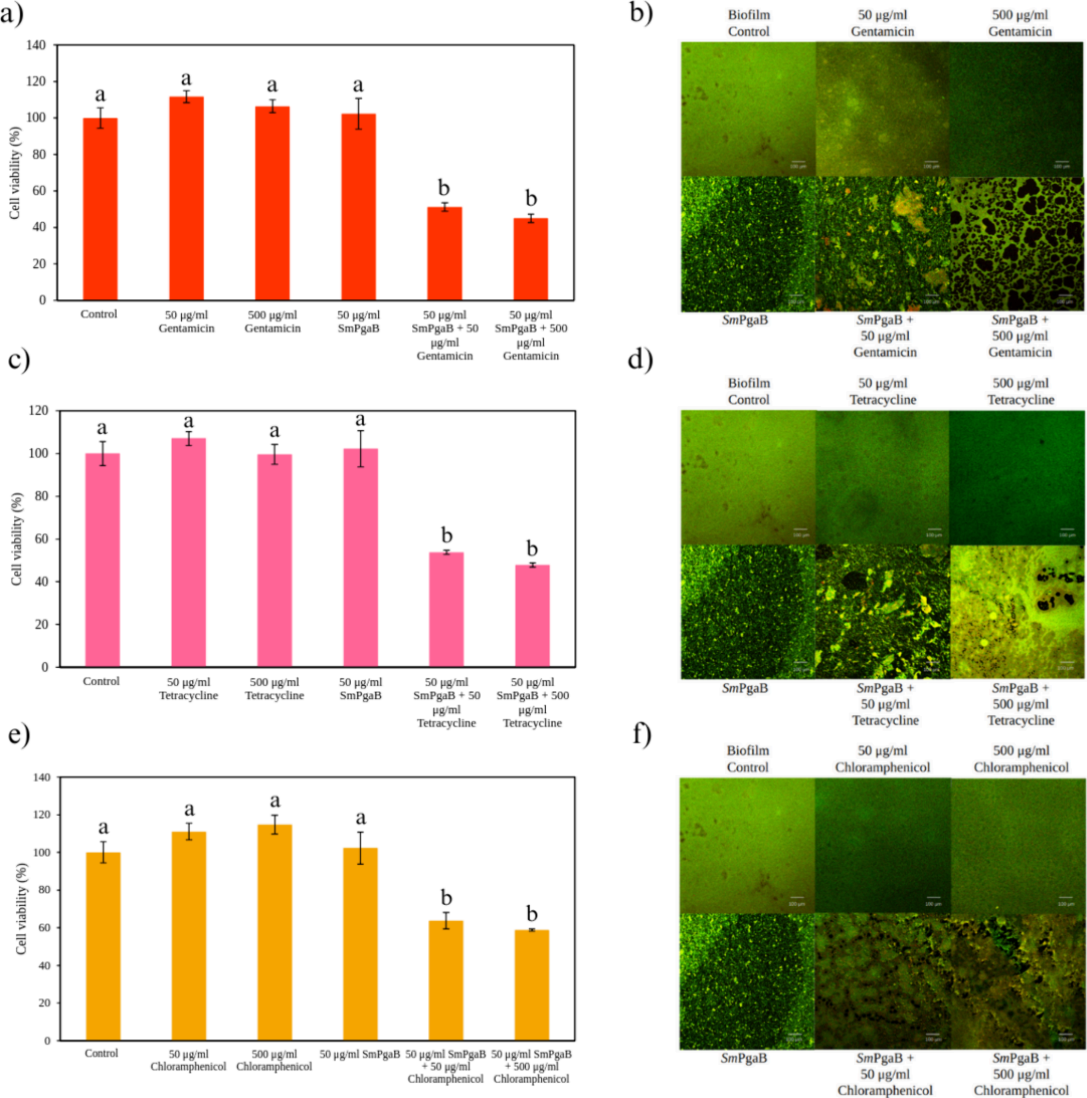
Synergism profile between *Sm*PgaB and (a) gentamicin; (c) tetracycline; (e) chloramphenicol. Confocal laser scanning microscopy of the *S. aureus* 3 BF after and before the treatment with 50 μg/mL of *Sm*PgaB of (b) gentamicin; (d) tetracycline; (f) chloramphenicol. All antibiotics were analyzed at concentrations of 50 and 500 μg/mL in a 24 h biofilm and incubated for 24 h at 37 °C before the resazurin fluorescence test or LIVE/DEAD CSLM assay. The results were analyzed using a one-way ANOVA followed by Tukey’s HSD test (*n* = 6), which identified significant differences between the control and treatment groups (*p* < 0.01). Bars labeled with the same letter indicate *p* > 0.05, signifying no significant difference between those treatment pairs.

As a next step, we conducted CSLM imaging of *S. aureus* BFs. The comparison of the effect of antibiotics on the *S. aureus* 3 biofilm, as evidenced by the CSLM study, reveals that the BF is homogeneous with small cluster regions as observed by the Calcofluor White and Sypro Ruby assays. In addition, the enzyme action alone altered the BF architecture and promoted the detachment of the majority of the dead cells, which were no longer adhered to the remaining BF (Figure 10b,d,f). When applied alone, in both concentrations studied, the antibiotics cannot penetrate the BF, affecting its superficial layer solely. However, as a combination with the enzyme, an enhanced combined effect can be seen, which is in accordance with results from refs 35 and 36; as in the regions where the PNAG of the BFs was degraded by the enzyme, there is an increased DEAD signal. LIVE/DEAD fluorescent assays of each studied strain BF (Supporting Information Figures S11–S13) further support the notion of increased efficiency of tested antibiotics in the presence of *Sm*PgaB.

## Conclusions

Here, we describe the biochemical and structural characterization of PgaB enzyme from *Serratia marcensens* (*Sm*PgaB). Our results confirm that *Sm*PgaB has a two-domain architecture containing both a CE4 domain and a GH153 hydrolytic domain. Furthermore, the *Sm*PgaB enzyme showed high BF degradation potential (up to 92%) when applied to poly-β-1,6-*N*-acetyl-d-glucosamine-rich *Staphylococcus aureus* BFs. The enzyme also has a high inhibition potential of *S. aureus* BFs. Moreover, when associated with the antibiotics gentamicin, tetracycline, and chloramphenicol, SmPgaB enhanced their antimicrobial capacity, exhibiting a reduction of viable cells when applied to the BF alongside the antibiotics. Confocal laser scanning microscopy also demonstrated changes in the BF morphology after treatment. These results provided insights into applications of *Sm*PgaB β-1,6-*N*-acetylglucosaminidase for hydrolytic degradation of PNAG-rich *S. aureus* biofilms and boosting antibiotic efficiency by such degradation.

## Material and Methods

### Genomic Sequencing of the *S. aureus* Isolates

*S. aureus* isolates SA1, SA2, and SA3 have been obtained and characterized as described previously.^17^ The sequencing reads from the genomes of *S. aureus* isolates SA1 (*S. aureus* 1, NCBI Biosample SAMN49625301), SA2 (*S. aureus* 2, NCBI Biosample SAMN49625302), and SA3 (*S. aureus* 3, NCBI Biosample SAMN49625303) were submitted to genome assembly using Unicycler.^37^ The reference genome of *S. aureus* (RefSeq ASM1672758v1) was used to refine the assemblies via RagTag,^38^ which were then annotated using Prokka^39^ and evaluated using QUAST.^40^

Circular diagrams representing the genomes of the *S. aureus* 1, 2, and 3 were generated using the circlize package (v0.4.16) and default settings.^41^ Processing of structural annotation of coding genes based on the GFF files of the mentioned genomes was performed using default settings of the rtracklayer package (v1.66.0),^42^ considering the data segregation by strand orientation (sense or antisense). A sliding window (1,000 bp size) was used throughout the genomes to calculate the GC content to identify genomic variability regions using the Biostrings package (v2.74.1).^43^

Synteny analysis was performed to identify gene order conservation and structural rearrangements among *S. aureus* 1, 2, and 3 isolates using the genoPlotR package (v0.8.11).^44^ Homologous regions were defined by local alignment (BLASTn), applying quality filters to exclude artifacts or fragments of less than 2 Kb in size; the same analysis was performed, restricting fragments to those of greater than 5 Kb.

Characterization of the ica operon (icaADBC) to identify polymorphisms was based on a multiple sequence alignment of the promoter region (upstream regulatory sequence with 300 bp size) using the MUSCLE algorithm wrapped in package msa (v1.38.0).^45^ Scanning for single-nucleotide polymorphism and graphical visualization of nucleotide matrices were performed using the packages ggplot2 (v3.5.2) and reshape2 (v1.4.5). Furthermore, the coding sequences of the icaA, icaB, icaC, and icaD genes were translated *in silico*. The amino acid chains were further aligned, and nonsynonymous substitutions were searched.

### SmPgaB Biochemical Characterization

Phylogenetic analysis, cloning, expression, and purification procedures of the enzyme are described in the Supporting Information. Since *Sm*PgaB has no activity against the synthetic substrate 4-nitrophenyl-*N*-acetyl-β-d-glucosaminide, the optimal pH, temperature, thermal stability, and residual activity assays were carried out using a PNAG-rich *S. aureus* BF as a substrate. For these assays, *Staphylococcus aureus* 3 BF growth and crystal violet (CV) staining were conducted. After biofilm maturation, the BF was washed thrice with a 0.9% (w/v) NaCl solution. For the optimal pH assay, it was treated with 50 μg/mL *Sm*PgaB and 20 mM citrate-phosphate buffer ranging from pH 2.0 to 8.0 and incubated at 37 °C for 4 h. The pH stability was then measured by quantifying the remaining biofilm biomass using the CV assay. The absorbance of the eluted dye at 595 nm corresponds to the amount of nondegraded biofilm, with lower absorbance indicating higher enzymatic activity at that pH. After the optimum pH was determined, the thermal stability tests were performed using 20 mM citrate-phosphate buffer pH 6 and 50 μg/mL *Sm*PgaB that were submitted to an hour of incubation in a temperature range of 20–90 °C before being applied in the BF. Residual activity assays were carried out, with temperatures of 40 and 70 °C over a period of 24 h using 20 mM citrate-phosphate buffer pH 6 and 50 μg/mL *Sm*PgaB. All assays were done in sextuplicate.

### Thermal Shift Assays

The effect of different buffers and pH conditions was evaluated by differential scanning fluorimetry (Thermofluor).^46,47^ 48 different buffers were analyzed by combining 5 μL of the enzyme at 28 μM with 10 μL of each buffer and 5 μL of 2000 × SYPRO Orange dye (Invitrogen; California, USA), 300 times diluted in water. The samples were incubated in an iCycler iQ Real-Time PCR Detection System (Bio-Rad, USA) with a temperature range of 25 to 90 °C with stepwise increments of 1 °C per min and 10 s hold step. Fluorescence intensity was monitored with excitation/emission wavelengths at 490/530 nm, respectively, and the melting temperatures (*T*_m_) were recorded.

### SmPgaB 3D Structure Prediction and Binding Site Analysis

The three-dimensional model of *Sm*PgaB was obtained using the AlphaFold deep learning prediction algorithm.^48^ Local alignments were performed with BLAST using PDB as the source database, and the closest homologue with available crystallographic structure, preferentially containing ligands in the catalytic region, was selected for comparison. In order to identify the ligand binding site, *Sm*PgaB was superimposed with the crystallographic structure of a poly-β-1,6-*N*-acetylglucosaminidase from **Escherichia coli* K-12* bound to nickel (*Ec*PgaB, 4F9D) for the complete structure, the CE4 *Sm*PgaB subunit was compared to the CE4 subunit of **Vibrio cholerae* O1 biovar El Tor str. N16961* (4OUI and 4NZ1) complex with a NAG polymer, and the GH153 *Sm*PgaB domain was compared with the *Ec*PgaB GH153 domain complexed with a NAG polymer (PDB id: 4P7Q) and PNAG hexamer (PDB id: 4P7R). Superimposition, structural visualization, and figure elaboration were performed with PyMOL (PyMOL Molecular Graphics System, Version 3.1.0, Schrödinger, LLC). The predicted structure was used as the target in docking studies using SwissDock,^49^ and the ligand conformation was obtained from the PDB of *Ec*PgaB complexed with a PNAG hexamer (4P7R).

### *S. aureus* Isolates Biofilm Eradication and Growth Inhibition

To analyze the eradication potential of *Sm*PgaB when applied to plate-adhered BFs, three different clinical strains of *Staphylococcus aureus* were used. To grow *S. aureus* BFs, the strains were plated in Luria–Bertani agar media and grown at 37 °C for 24 h. Next, one colony was transferred to 5 mL of tryptic soy broth (TSB) supplemented with 0.75% (w/v) glucose and grown overnight at 37 °C under static conditions. Following said period, the liquid culture was diluted with TSB supplemented with 0.75% (w/v) glucose until its OD_600_ = 0.1. Afterward, 200 μL of the inoculated medium was transferred to 96-well plates and grown for another 24 h at 37 °C.

After the BF formation, the supernatant was discarded, and the BF was cleaned of any additional cells by washing thrice with a 0.9% (w/v) NaCl solution, and underwent an enzymatic treatment of *Sm*PgaB in concentrations of 50, 10, 1, and 0.1 μg/mL and 20 mM of phosphate-citrate buffer pH 6 for 4 h at 37 °C. After the treatment, the residual BF was washed thrice with a saline solution and stained with 0.1% (w/v) of crystal violet for 5 min. Subsequently, the BF was destained with 30% (v/v) acetic acid, and the solution's absorbance was measured at 595 nm in an Infinite 200 M PRO microplate reader (Tecan, Hombrechtikon, Switzerland). To calculate the reduction of BF biomass after treatment, the following equation was used:

$$\%\;\mathrm{Biofilm}\;\mathrm{biomass}=\frac{{\mathrm{Sample}}_{595}}{{\mathrm{CG}}_{595}}\times 100\%$$ 1

in which Sample_595_ is the absorbance of each treated well, and CG_595_ is the absorbance found for the control group. Each treatment was done in sextuplicate.

For the inhibition assays, the inoculated liquid culture was added to 96-well plates alongside *Sm*PgaB in concentrations of 50, 10, 1, and 0.1 μg/mL and incubated at 37 °C for 24 h under static conditions. The resulting BF was then stained following the same procedure as described above and quantified using eq 1.

For a qualitative imaging of the BF degradation capacity of *Sm*PgaB, the *S. aureus* 3 isolate BF was studied by confocal laser scanning microscopy (CLSM), using FilmTracer SYPRO Ruby Biofilm Matrix and Calcofluor White stains, which are specific for proteins and carbohydrates, respectively. After the BF had grown following the procedure previously described, the remnant planktonic cells were discarded, and the BF was rinsed three times with ultrapure water. Five hundred microliters of SYPRO Ruby were applied to the BF, and it was allowed to incubate for 20 min in the dark at room temperature. Then, the BF was washed with 0.9% (w/v) NaCl to remove excess dye, stained with 500 μL of Calcofluor White, incubated for 15 min in the dark, and subsequently washed with 0.9% (w/v) NaCl to remove excess stain. Qualitative data acquisition was conducted using a Zeiss LSM 780 confocal microscope (Zeiss, Oberkochen, Germany). The fluorescence was detected in the wavelengths of excitation/emission 405/563 and 458/599 nm for Calcofluor White and SYPRO Ruby, respectively.

### Synergism of the Enzyme with Antibiotics

For the synergy assays of the target enzyme with antibiotics, the BFs were grown as described previously. After the BFs matured, the supernatant was discarded and washed thrice with a 0.9% (w/v) NaCl solution, and the BF was submitted to a treatment with 50 μg/mL of *Sm*PgaB together with 50 and 500 μg/mL of gentamicin, tetracycline, or chloramphenicol in a TSB medium supplemented with 0.75% (w/v) glucose.

For the viability assays, after the incubation period, the supernatant was discarded, the BF was cleaned thrice with PBS, and 200 μL of PBS was added to the biofilm and used to resuspend the remnant biofilm. A 40 μL aliquot of resazurin at 0.15 mg/mL of resazurin (Sigma-Aldrich, St. Louis, USA) was added to the resuspended BF, and the mixture was incubated for 2 h at 37 °C. After incubation, the fluorescence was measured at the excitation/emission wavelengths of 550/590 nm, respectively, in an Infinite 200 M PRO microplate reader (Tecan, Hombrechtikon, Switzerland). The results were expressed as cell viability relative to the control BF:

$$\%\;\mathrm{Cell}\;\mathrm{viability}=\frac{{\mathrm{Sample}}_{550/590}}{{\mathrm{CG}}_{550/590}}\times 100\%$$ 2

where Sample_550/590_ equates to the fluorescence of the sample, and CG_550/590_ is related to the fluorescence of the control group. Each assay was done in sextuplicate compared to the control BF.

Additionally, the viability and BF distributions were analyzed by confocal laser scanning microscopy (CLSM). For this, the grown BF was washed three times with ultrapure water and stained using a fluorescence-based LIVE/DEAD assay (Sigma-Aldrich, St. Louis, USA). The BF was stained with a mixture of acridine orange (dead cells) and ethidium bromide (live cells). Qualitative data acquisition was done in a Zeiss LSM 780 confocal microscope (Zeiss, Oberkochen, Germany). The fluorescence was detected in the wavelengths of excitation/emission 415/540 and 580/620 nm for acridine orange and ethidium bromide, respectively.^50^

### Statistical Analysis

The results shown are given with their respective standard deviations (SD). Statistical analysis has been performed using a one-way analysis of variance (one-way ANOVA), followed by Tukey’s HSD post hoc test for pairwise comparisons among treatment groups. Results were considered statistically relevant at p-values less than 0.01 (*ps* < 0.01).

## Supplementary Material



## Notes

Phylogenetic trees of GH153 and CE4 proteins (Figures S1–S3); SDS-PAGE analysis of purified SmPgaB (Figure S4); structural stability analysis including thermal shift assays and circular dichroism spectra (Figure S5); sequence conservation and surface property mapping (Figure S6); SEC-SAXS experimental parameters and fitting statistics (Table S1); SEC-MALS chromatograms and molecular weight determination in solution (Figure S8); additional molecular modeling and ensemble analysis (Figure S9); comparative enzymatic hydrolysis of biofilms with *Ka*PgaB, papain, and DNase (Figure S10); LIVE/DEAD fluorescence assays for antibiotic synergy across all studied strains (Figures S11–S13); and detailed procedures for phylogenetic analysis, cloning, expression, and purification (PDF)
