# Supplementary material for: Biochemical and Structural Characterization of Two-domain Glycoside Hydrolase PgaB from Serratia marcescens and Its Application for S. aureus Biofilm Degradation
Source: ACS Infect Dis. 2026 Jun 15;12(7):2262–76. doi: 10.1021/acsinfecdis.6c00086 (PMC13366581; doi:10.1021/acsinfecdis.6c00086)
Supplement: Supplementary file 1 [file id6c00086_si_001.pdf]

Supporting information

Biochemical and Structural Characterization of Two-domain Glycoside Hydrolase PgaB from *Serratia marcescens* and its Application for *S. aureus* Biofilm Degradation

Amanda Freitas Cruz[1], Pedro Ricardo Vieira Hamann[1], Francisco Eduardo Gontijo Guimaraes[1], Andrei Nicoli Gebieluca Dabul[2], Ruth Celestina Condori Mamani[1], Marcos Pileggi[3], Mario de Oliveira Neto[4], Matheus Rodrigues Sauda[5], Guilherme Targino Valente[6], Tsutomu Matsui[7], Thomas M. Weiss[7], Evandro A. Araújo[8] & Igor Polikarpov[1, *]

[1]Instituto de Física de São Carlos, Universidade de São Paulo, Avenida Trabalhador São-carlense 400, 13566-590 São Carlos, SP, Brazil

[2]School of Pharmaceutical Sciences, São Paulo State University, Rodovia Araraquara-Jáu, km 1, 14800-903, Araraquara, SP, Brazil

[3]Environmental Microbiology Laboratory, Life Sciences and Health Institute, Structural and Molecular Biology, and Genetics Department, Ponta Grossa State University, Ponta Grossa, Brazil.

[4]Institute of Biosciences, Sao Paulo State University, District of Rubiao Jr., 18618-970, Botucatu, SP, Brazil

[5]Laboratory of Applied Biotechnology, São Paulo State University, Botucatu, 18618-687, Brazil

[6]Clinical Hospital of Medical School of Botucatu, Botucatu, 18618-687, Brazil

[7]Stanford Synchrotron Radiation Lightsource, SLAC National Accelerator Laboratory, 2575 Sand Hill Rd, Menlo Park, CA 94025, USA

[8]Brazilian Synchrotron Light Laboratory, Brazilian Center for Research in Energy and Materials, Giuseppe Maximo Scolfaro, 10000, Campinas, SP 13083-970, Brazil

[*]Corresponding author, e-mail: ipolikarpov@ifsc.usp.br

Supplementary results

Phylogenetic analysis

Full-length *Sm*PgaB has 668 amino acid residues and its fold is characterized by two different domains: carbohydrate esterase family 4 (CE4) N-terminal domain (amino acid residues 17 to 309), and a GH153 domain comprising amino acid residues 310 to 668. To better comprehend the evolutionary variations of *Sm*PgaB, we built a phylogenetic tree including both uncharacterized and characterized GH153 members.

Phylogenetic analysis of GH153 and CE4 protein sequences from UniProt revealed six major sequence clusters, with *Serratia marcescens* represented within this structure (Supplementary Fig. S1). The analysis provided insights into the evolutionary context and functional relationships of *Serratia marcescens* GH153/CE4 homologs among diverse bacterial taxa. The analysis showed 6 main clusters of these enzymes. Cluster 2 (85 members), which includes *Serratia marcescens*, was the largest and primarily composed of *Pseudomonas* species, indicating a broad but conserved group of gamma-proteobacteria. The inclusion of *S. marcescens* within this cluster suggests a close evolutionary relationship in terms of gene sequence and domain architecture, particularly within CE4 family deacetylases. The shared clustering implies either vertical inheritance of conserved functional domains or possible horizontal gene transfer events within ecologically flexible genera (Supplementary Fig. S2). It revealed three principal clades, corresponding to core *Pseudomonas* (red), the aquatic group (blue), and soil bacteria (green). The core *Pseudomonas* clade, comprising two species, exhibited the shortest branch lengths, indicating a minimal evolutionary distance and suggesting strong conservation of the GH153 and CE4 genes within this lineage.

The cluster 1 (5 members) included halotolerant taxa such as *Halomonas* spp. and *Vreelandella songnenensis*, representing a small, well-defined clade distinct from *S. marcescens* in both ecological adaptation and sequence lineage. This group likely reflects niche-specific functional specialization unrelated to the patho-ecological traits of *S. marcescens*. Cluster 3 (3 members) grouped *Halioxenophilus aromaticivorans*, *gamma-proteobacterium HTCC5015*, and an unclassified *Pseudomonadota* species. These sequences showed weak phylogenetic proximity to *S. marcescens*, likely representing a more basal or divergent lineage. Cluster 4 (37 members) included diverse genera such as *Ralstonia*, *Variovorax*, and *Neoroseomonas*. While taxonomically broader, this group exhibited some structural similarities in CE4 domains. However, *Serratia marcescens* was more distantly related to members of this cluster compared to those in cluster 2. Cluster 5 (10 members) comprised methanotrophs such as *Methylomonas* and *Methylococcus*. Their GH153 and CE4 homologs displayed low sequence similarity to *S. marcescens*, indicating divergent functional evolution. Cluster 6 (9 members) grouped ecologically diverse genera like *Chromobacterium*, *Aquitalea*, and *Acinetobacter*. Although scattered in taxonomic origin, several members showed moderate sequence similarity with *S. marcescens*, potentially reflecting conserved domains adapted to generalist bacterial lifestyles. *S. marcescens* PgaB gene is positioned within a large, functionally rich cluster alongside *Pseudomonas* species, indicating the conserved yet versatile nature of GH153 and CE4 enzymes within this clade.

In addition, a phylogenetic analysis was conducted using GH153 and CE4 protein sequences from the CAZy database that were previously characterized. The resulting tree revealed three principal clusters, reflecting evolutionary relationships among bacterial and fungal taxa (Supplementary Fig. S3). Cluster 1 consisted of six bacterial sequences from *Aeromonas*, *Staphylococcus*, *Ammonifex*, *Bordetella*, *Serratia marcescens*, and *Escherichia*. The relatively tight grouping of these species

suggests that despite belonging to diverse bacterial lineages, the GH153 and CE4 proteins maintain a conserved structural core. The presence of *Serratia marcescens* within this cluster indicates its evolutionary proximity to clinically relevant and environmental bacteria, reflecting conserved functions that may be essential across varied ecological niches. Cluster 2, encompassing 35 members, was predominantly composed of sequences from *Vibrio*, *Streptomyces*, and various fungal genera, including *Schizophyllum*, *Phycomyces*, *Gongronella*, *Amylomyces*, and *Rhizopus*. Cluster 3 contained a single member, the GH153 or CE4 protein from *Schizosaccharomyces*. Overall, the phylogenetic structure highlights the broad evolutionary distribution of GH153 and CE4 enzymes, with *Serratia marcescens* positioned within a conserved bacterial clade. The *Sm*PgaB gene is conserved within a conserved bacterial clade, especially with *Aeromonas*, *Staphylococcus*, *Ammonifex*, *Bordetella*, and *Escherichia* enzymes, distinguishing *S. marcescens* from more divergent fungal and aquatic bacterial lineages.

Supplementary material and methods

Phylogenetic analysis

Protein sequences were retrieved from the UniProt Knowledgebase (accessed April 2025) and CAZY database (accessed April 2025) for representative taxa spanning bacterial and fungal lineages of GH153 and CE4 enzymes family. Redundant sequences were removed using CD-HIT (v4.8.1) with a 35-95% identity threshold.

To investigate the evolutionary relationships of GH153 and CE4 protein sequences, a phylogenetic tree was constructed based on a curated set of sequences derived from the UniProt database. The

multiple sequence alignment was first generated using MUSCLE[1], and the resulting alignment was used to construct a Newick-formatted tree via a custom Python 3.10 scripts.

Hierarchical clustering was performed using SciPy (v1.8.1) with UPGMA linkage, a 0.4 distance threshold determined by knee-point detection, and branch optimization via Neighbor-Joining algorithm (Bio.Phylo v1.79). The phylogenetic tree was visualized using Matplotlib (v3.7.1) with bootstrap-supported branch widths and color-coded clades. Cluster robustness was validated through 1000 bootstrap replicates. The computational scripts (NumPy v1.23.5, SciPy v1.8.1) are available upon request.

Cloning, expression and purification

The coding sequence of *Serratia marcescens subsp. marcescens* (*Sm*PgaB) (ATCC 13880; GenBank access number QTI64407.1) was amplified as described in (Camilo & Polikarpov, 2014)[2]. The genomic amplification of S*m*PgaB was done by polymerase chain reaction (PCR) with 5 µL 5X Phusion HF Buffer (New England BioLabs), 0.5 µL 10 mM dNTPs, 1.25 µL of forward (5' CAGGGCGCCATGTGCAGTCAGGCGGACG 3') and reverse (5' GACCCGACGCGGTTATCATGGCAGTGGGAACC 3') primers at 10 µM, 25 ng of genomic DNA, 0.5 µL of Phusion DNA Polymerase (New England BioLabs), and volume adjusted to 25 µL with PCR grade water. The resulting pETTRXA-1a/LIC expression vector with the target gene was transformed by a heat shock method into competent *Escherichia coli DH5α* and these were used for plasmid propagation and overexpressed using *E. coli* BL21 (DE3) for *Sm*PgaB$^{18\text{-}668}$.

The heterologous production of *Sm*PgaB was performed by cultivating *E. coli* BL21 (DE3) containing the construct pETTRXA-1a/LIC-*Sm*PgaB in autoinduction medium[3] with 50 µg/mL kanamycin, at 28 °C and 200 rpm agitation per 24h. The cells were harvested by centrifugation at

13 000 x g at 4 °C for 20 min, resuspended in 50 mM Tris-HCl pH 8.5 (containing 300 mM NaCl, 10 mM imidazole, and 0.1 mM phenylmethylsulfonyl fluoride) and sonicated in an ice bath on a 550 Sonic Dismembrator Sonifier (Fisher Scientific, Hampton, USA) with 40% of amplitude in seven cycles of 30 s on and off. After cell disruption, the supernatant was clarified by centrifugation at 13 000 x g for 20 min at 4 °C.

The clarified supernatant was loaded onto a His-Trap column in an Akta Purification system, and the unbounded protein fraction was washed out with 10 column volumes (CV) of 50 mM Tris-HCl pH 8.5, 300 mM NaCl and 10 mM imidazole, followed by an isocratic elution with 50 mM Tris-HCl pH 8.5, 300 mM NaCl and 500 mM imidazole. After extensive dialysis in water to remove the purification buffer, the resultant recombinant protein was incubated in the presence of TEV protease in a proportion of 5:1 of target enzyme to TEV and 5 mM of dithiothreitol for 24 h to cleave the His-Trx tag and then re-loaded onto a buffer-equilibrated Ni-NTA agarose resin.

Enzyme purity was evaluated with SDS-PAGE 12%. Protein concentration was quantified using NanoDrop 2000 Spectrophotometer (Thermo Scientific, Waltham, USA) at 280 nm using its extinction coefficient (ε = 133 730 M^{-1} cm^{-1}) and its theoretical mass (73.292 kDa) (Supplementary Fig. S7).

Dynamic light scattering assays

Dynamic light scattering (DLS) assays for both proteins were carried out in a Malvern ZetaSizer Nano series Nano-ZS (model ZEN3600) instrument (Malvern Zetasizer, Worcestershire, UK) equipped with a 173° scattering angle, with a He-Ne laser λ = 633 nm, at 25 °C. For the assay of polydispersity as a function of salt concentration, the enzyme was used at a concentration of 12

μM with 10 mM of NaCl in the range of 0 to 300 mM and incubated for 2 h. As for the assay of the enzyme's polydispersity in function of pH, the protein was used at the same concentration of the previous assay, it was incubated for 2 h with 10 mM of sodium acetate (pH 4.5-5) and potassium phosphate (pH 6-8) to achieve the desired pH. The sample volume used for analysis was 78 μL, and the measurements were done in a quartz glass cell. All samples were centrifuged to avoid interference of larger particles. An average of 15 scans with a duration of approximately 5 min were done for each sample. All measurements were done in triplicate (Supplementary Fig. S8).

The scattering intensity data was processed using the Zetasizer (version 8.02) software to encounter the hydrodynamic diameter (D_H) from the Stokes-Einstein equation[4]:

$$D = \frac{kT}{6\pi\eta R_H} \qquad (S1)$$

in which, D is the diffusion coefficient, k is the Boltzmann constant, and η is the viscosity of the solution. Additionally, the size distribution of scatterers is also found in each sample[5] . This is obtained from the autocorrelation function of the time-dependent fluctuation of the intensity of light scattered from the particles in solution[6] (Supplementary Fig. S8).

Size-exclusion chromatography coupled with multi-angle light scattering

Size-exclusion chromatography coupled with multi-angle light scattering (SEC-MALS) was used to determine the molecular mass and oligomerization state of the recombinant *Sm*PgaB[7–9]. The samples were subjected to size-exclusion chromatography using a WTC030N5 column (Wyatt Technology, Santa Barbara, USA) coupled to a HPLC system equipped with a miniDAWN

TREOS multi-angle light scattering detector (Wyatt Technology, Santa Barbara, USA) and a Optilab T-rEX refractometer (RI) (Wyatt Technology, Santa Barbara, USA).

The protein sample was concentrated to 2 mg/mL using a 10 kDa molecular weight cutoff centrifugal filter (Millipore, Burlington, USA) before analysis. The sample was centrifuged at 10,000 x g for 20 min at 4 °C to avoid the presence of aggregates and larger particles. The buffer used for the analysis was PBS pH 7.4 and all data was collected at room temperature (25 ° C) (Supplementary Fig. S9).

To ensure accuracy, the MALS detector was calibrated using a monodisperse bovine serum albumin (BSA) standard, in the same buffer as the samples[10]. Data collection and SEC-MALS analysis were performed with the ASTRA 8 software (Wyatt Technology, Santa Barbara, USA). The molecular mass was calculated using the Zimm formalism, which combines light scattering intensity with concentration derived from the RI detector. The dn/dc (refractive index increment) value for all samples was defined as 0.185 mL/g[11] and for solvents was 1.328.

Circular Dichroism analysis

Circular dichroism (CD) measurements were carried out using a J-815 CD Spectropolarimeter (JASCO Corporation, Tokyo, Japan) equipped with a temperature control device in the Far-UV region (200-260 nm) and the Spectra Manager II software (Jasco). *Sm*PgaB was used in the concentration of 12 μM with 10 mM sodium acetate (pH 3.6-5) and potassium phosphate (pH 6-8) and incubated overnight. All data was collected at 20 °C using a 1 mm quartz cuvette, the CD spectrum was obtained by signal averaging of 10 spectra with 1 s response. The protein spectrum was found by subtracting the buffer spectrum from the sample spectrum. All samples were

centrifuged to avoid any interference from particles in the suspension. The final analysis was done using the single analysis mode of the BeStSel method (https://bestsel.elte.hu/).

For the measurements of the thermal denaturation curve, *Sm*PgaB was used at the same concentration of the previous experiment with 10 mM potassium phosphate pH 6 which were incubated overnight. After incubation, the protein was centrifuged. For data acquisition, a 1 mm quartz cuvette was used. The temperature was set at 20 °C and increased by 1 °C per min reaching 90 °C. The temperature was held with a maximum fluctuation of 0.1 °C.

The evaluation of the secondary structure of *Sm*PgaB was performed by the circular dichroism (CD). The enzyme has a CD spectrum similar to those of predominantly α-helical proteins, having minimum peaks at the region of 205-222 nm[12,13]. Moreover, when the pH of the enzyme solution was varied, the form of spectrum remains similar, presenting only a small displacement of the curves especially in the minimum at 222 nm, which indicates that the α-helices maintain the protein stability (Supplementary Fig. S1b). Next, the analysis of the spectrum by the BeStSel method [14] showed that 50% of the structure of *Sm*PgaB is composed of α-helices, 33% equates to β-sheets and the remaining 17% consisting of loops and other structures. In addition to the pH study, the thermal denaturation of the enzyme was studied at the wavelengths of 205, 208, 212, 215, 218, 222, and 225 nm (Supplementary Fig. S1c). The experimentally measured melting temperature of *Sm*PgaB was 59.1 ± 0.4 °C a, which is similar to that found through the thermal shift assays, indicating that the enzyme during a process of thermal denaturation loses their secondary and tertiary structure, simultaneously.

Polydispersity and oligomerization state

The effect of NaCl concentration and pH on *Sm*PgaB stability was observed through its polydispersity and hydrodynamic radius found by dynamic light scattering assays. The size distribution graphs of the enzyme (Supplementary Fig. S8a) presented a sole, symmetric peak indicative of a monodisperse population. Furthermore, the polydispersity index (PDI) calculated showed that *Sm*PgaB maintained a low polydispersity throughout all conditions tested (Fig. S8b, c), suggesting high structural integrity.

*Sm*PgaB exhibited a decreased PDI in all analyzed conditions in the presence of salt (Supplementary Fig. S9b). At lower NaCl concentration, *Sm*PgaB maintained an average polydispersity below 30%. At high ionic strength (≤ 50 mM), the enzyme presented stable polydispersity values converging around 30-40%. Regarding the pH (Supplementary Fig. S8c), *Sm*PgaB demonstrated stability over the conditions evaluated, especially in the range of pH 6.5-7.5 maintaining a PDI close to 30% and having a minimum PDI of 18% at pH 7.0.

The average hydrodynamic radius, obtained by the Zetasizer software, for *Sm*PgaB across all conditions was 3.73 ± 0.05 nm. The analysis of *Sm*PgaB across distinct NaCl concentrations and pH conditions revealed consistently low polydispersity values, indicating a high degree of structural stability. This suggests that *Sm*PgaB maintains a well-defined conformational ensemble under varying solution conditions. The observed stability may be associated with the presence of disordered regions at both the N- and C-termini, which can contribute to structural adaptability while preserving the integrity of the folded core.

To assert the oligomerization and molecular weight of the enzymes in solution, SEC-MALS was applied. This technique allows for the determination of absolute molar mass and size of proteins in solution[15]. Experiments were performed in a PBS buffer (10 mM Na_2HPO_4, 1.8 mM KH_2PO_4,

137 mM NaCl, 2.7 mM KCl pH 7.4) with the enzyme at a concentration of 2 mg/mL and the results were given by the differential refractive index (DRI) and molecular weight (MW) found by the Multi-Angle Light Scattering detector.

*Sm*PgaB had its main peak around 13-15 min, having a molecular weight of 71.7 ± 0.3 kDa (Supplementary Fig. S9). This result is closely related to the expected molecular weight of the monomeric state for *Sm*PgaB, indicating that the protein mainly exists as a monomer. However, a minor species is observed at ~12.7 min, with its relative abundance of 1% at 2 mg/mL. Additionally, its estimated molecular weight was 107 ± 5 kDa. This suggests the presence of oligomeric species aggregation, potentially dimers, higher-order aggregates.

Size-exclusion chromatography small angle X-ray scattering data collection and analysis

Size-exclusion chromatography small-angle X-ray scattering (SEC-SAXS) experiments were performed at the Stanford Synchrotron Radiation Light Source (SSRL) beamline BL4-2 [16] . After protein purification, the enzyme at a concentration of 8 mg/mL, was centrifuged at 17 000 g for 2h at 4 °C and later injected into a previously equilibrated with a PBS buffer (10 mM Na_2HPO_4, 1.8 mM KH_2PO_4, 137 mM NaCl, 2.7 mM KCl pH 7.4) Superdex 200 Increase PC 3.2/300 column (Cytiva, Marlborough, USA) integrated to a UHPLC (ThermoFisher UltiMate 3000) at a flow rate of 0.05 mL/min and images were collected with 1 s exposure time. The SEC-SAXS data was collected at a room temperature of 20 °C with q-range of 0.006-0.504 $Å^{-1}$.

The automated real-time data processing was done with the SECPipe software (https://www-ssrl.slac.stanford.edu/smb-saxs/content/documentation-software-secpipe). Once the average buffer scattering curve was obtained, the background-subtracted curves could be found[16]. After

this, using the ATSAS software suite (ATSAS, Version 4.0.0, BIOSAXS GmbH[17]), the gyration radius (R_g) was estimated from the Guinier equation, the pair distribution function and the maximum particle dimension (D_{max}) were obtained via the GNOM program[18]. *Ab initio* modeling was performed using DAMMIF[19], an average model was generated from 15 independent reconstructions using DAMAVER20 and the final model was improved using DAMMIN program[21]. The final model was superimposed to the AlphaFold-derived structure using the SUPCOMB program[22]. Finally, the SAXS-based molecular weights were estimated using SAXSMoW 2.0 (http://saxs.ifsc.usp.br/ [23]).

Flexibility analysis of *Sm*PgaB was performed utilizing experimental small-angle X-ray scattering (SAXS) intensity curves, employing the SREFLEX software[24]. Additionally, the conformational flexibility of the optimal model generated by SREFLEX was evaluated by rapid simulation-based modeling of protein structures using CABS-flex web server[25]. Moreover, the computed X-ray scattering curves were obtained in FOXS[26] or CRYSOL[27] programs.

Supplementary Figures

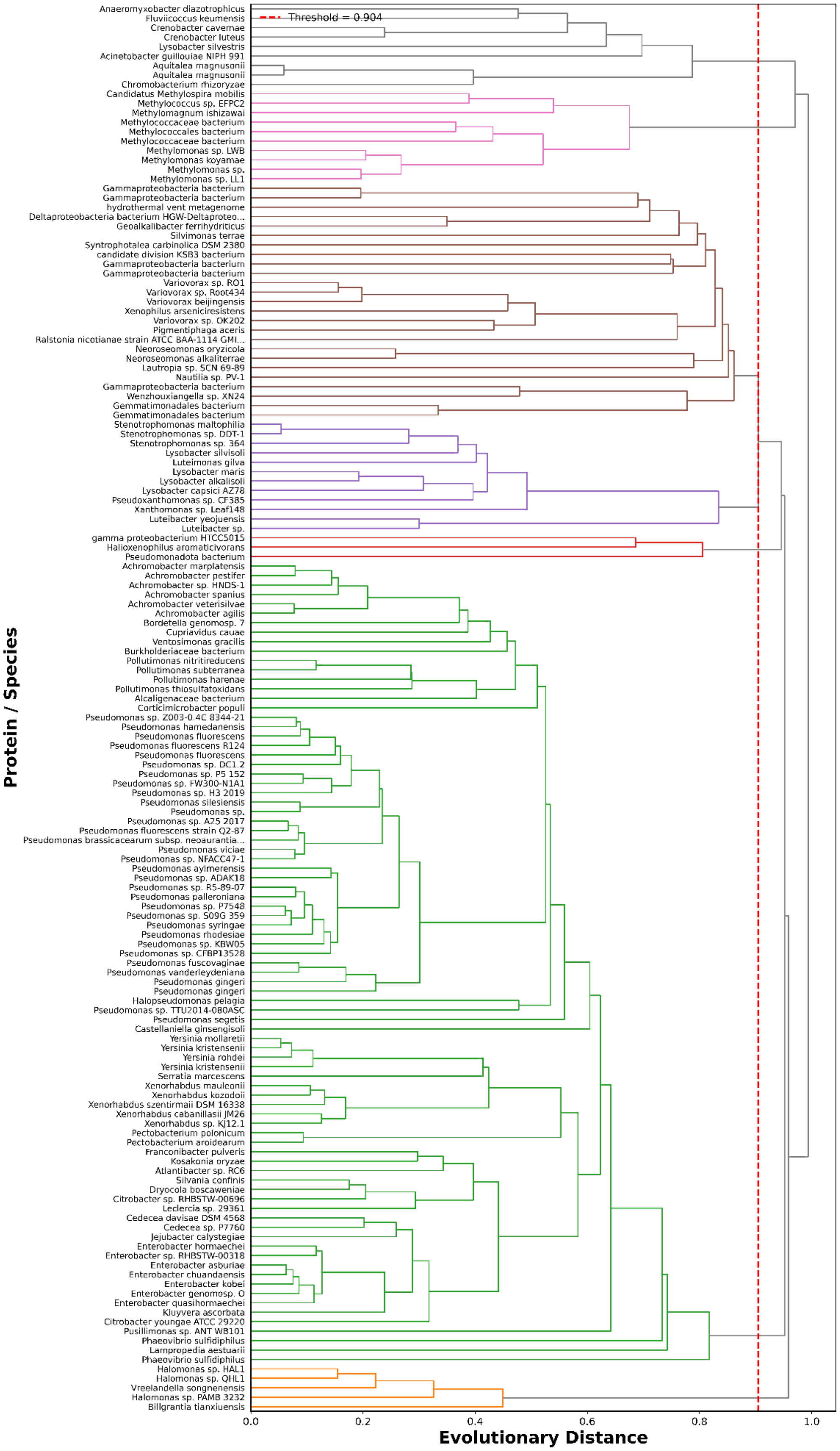
Threshold = 0.904
Anaeromyxobacter diazotrophicus
Fluviicoccus keumensis
Crenobacter cavernae
Crenobacter luteus
Lysobacter silvestris
Acinetobacter guillouiae NIPH 991
Aquitalea magnusonii
Aquitalea magnusonii
Chromobacterium rhizoryzae
Candidatus Methylospira mobilis
Methylococcus sp. EFPC2
Methylomagnum ishizawai
Methylococcaceae bacterium
Methylococcales bacterium
Methylococcaceae bacterium
Methylomonas sp. LWB
Methylomonas koyamae
Methylomonas sp.
Methylomonas sp. LL1
Gammaproteobacteria bacterium
Gammaproteobacteria bacterium
hydrothermal vent metagenome
Deltaproteobacteria bacterium HGW-Deltaproteo...
Geoalkalibacter ferrihydriticus
Silvimonas terrae
Syntrophotalea carbinolica DSM 2380
candidate division KSB3 bacterium
Gammaproteobacteria bacterium
Gammaproteobacteria bacterium
Variovorax sp. RO1
Variovorax sp. Root434
Variovorax beijingensis
Xenophilus arseniciresistens
Variovorax sp. OK202
Pigmentiphaga aceris
Ralstonia nicotianae strain ATCC BAA-1114 GMI...
Neoroseomonas oryzicola
Neoroseomonas alkaliterrae
Lautropia sp. SCN 69-89
Nautilia sp. PV-1
Gammaproteobacteria bacterium
Wenzhouxiangella sp. XN24
Gemmatimonadales bacterium
Gemmatimonadales bacterium
Stenotrophomonas maltophilia
Stenotrophomonas sp. DDT-1
Stenotrophomonas sp. 364
Lysobacter silvisoli
Luteimonas gilva
Lysobacter maris
Lysobacter alkalisoli
Lysobacter capsici AZ78
Pseudoxanthomonas sp. CF385
Xanthomonas sp. Leaf148
Luteibacter yeojuensis
Luteibacter sp.
gamma proteobacterium HTCC5015
Halioxenophilus aromaticivorans
Pseudomonadota bacterium
Achromobacter marplatensis
Achromobacter pestifer
Achromobacter sp. HNDS-1
Achromobacter spanius
Achromobacter veterisilvae
Achromobacter agilis
Bordetella genomosp. 7
Cupriavidus cauae
Ventosimonas gracilis
Burkholderiaceae bacterium
Pollutimonas nitritireducens
Pollutimonas subterranea
Pollutimonas harenae
Pollutimonas thiosulfatoxidans
Alcaligenaceae bacterium
Corticimicrobacter populi
Pseudomonas sp. Z003-0.4C 8344-21
Pseudomonas hamedanensis
Pseudomonas fluorescens
Pseudomonas fluorescens R124
Pseudomonas fluorescens
Pseudomonas sp. DC1.2
Pseudomonas sp. P5 152
Pseudomonas sp. FW300-N1A1
Pseudomonas sp. H3 2019
Pseudomonas silesiensis
Pseudomonas sp.
Pseudomonas sp. A25 2017
Pseudomonas fluorescens strain Q2-87
Pseudomonas brassicacearum subsp. neoaurantia...
Pseudomonas viciae
Pseudomonas sp. NFACC47-1
Pseudomonas aylmerensis
Pseudomonas sp. ADAK18
Pseudomonas sp. R5-89-07
Pseudomonas palleroniana
Pseudomonas sp. P7548
Pseudomonas sp. S09G 359
Pseudomonas syringae
Pseudomonas rhodesiae
Pseudomonas sp. KBW05
Pseudomonas sp. CFBP13528
Pseudomonas fuscovaginae
Pseudomonas vanderleydeniana
Pseudomonas gingeri
Pseudomonas gingeri
Halopseudomonas pelagia
Pseudomonas sp. TTU2014-080ASC
Pseudomonas segetis
Castellaniella ginsengisoli
Yersinia mollaretii
Yersinia kristensenii
Yersinia rohdei
Yersinia kristensenii
Serratia marcescens
Xenorhabdus mauleonii
Xenorhabdus kozodoii
Xenorhabdus szentirmaii DSM 16338
Xenorhabdus cabanillasii JM26
Xenorhabdus sp. KJ12.1
Pectobacterium polonicum
Pectobacterium aroidearum
Franconibacter pulveris
Kosakonia oryzae
Atlantibacter sp. RC6
Silvania confinis
Dryocola boscaweniae
Citrobacter sp. RHBSTW-00696
Leclercia sp. 29361
Cedecea davisae DSM 4568
Cedecea sp. P7760
Jejubacter calystegiae
Enterobacter hormaechei
Enterobacter sp. RHBSTW-00318
Enterobacter asburiae
Enterobacter chuandaensis
Enterobacter kobei
Enterobacter genomosp. O
Enterobacter quasihormaechei
Kluyvera ascorbata
Citrobacter youngae ATCC 29220
Pusillimonas sp. ANT WB101
Phaeovibrio sulfidiphilus
Lampropedia aestuarii
Phaeovibrio sulfidiphilus
Halomonas sp. HAL1
Halomonas sp. QHL1
Vreelandella songnenensis
Halomonas sp. PAMB 3232
Billgrantia tianxiuensis
0.0
0.2
0.4
0.6
0.8
1.0
Evolutionary Distance
Protein / Species

S14

Supplementary Figure S1: Hierarchical clustering of GH153 and CE4 sequences reveals six major clusters based on sequence similarity with protein sequence from Uniprot database. A dendrogram was generated using hierarchical clustering analysis to group previously characterized GH153 and CE4 protein sequences. Six distinct clusters were detected, as indicated by branch color separation. The clustering reflects varying degrees of sequence divergence, with closely related sequences showing shorter branch lengths and grouping together at lower distances. The red dashed line represents the distance threshold as 0.9045 used to define the clusters. This phylogenetic structure highlights the evolutionary relationships among a broad diversity of bacterial and fungal species, with *Serratia marcescens* positioned within one of the major bacterial clusters.

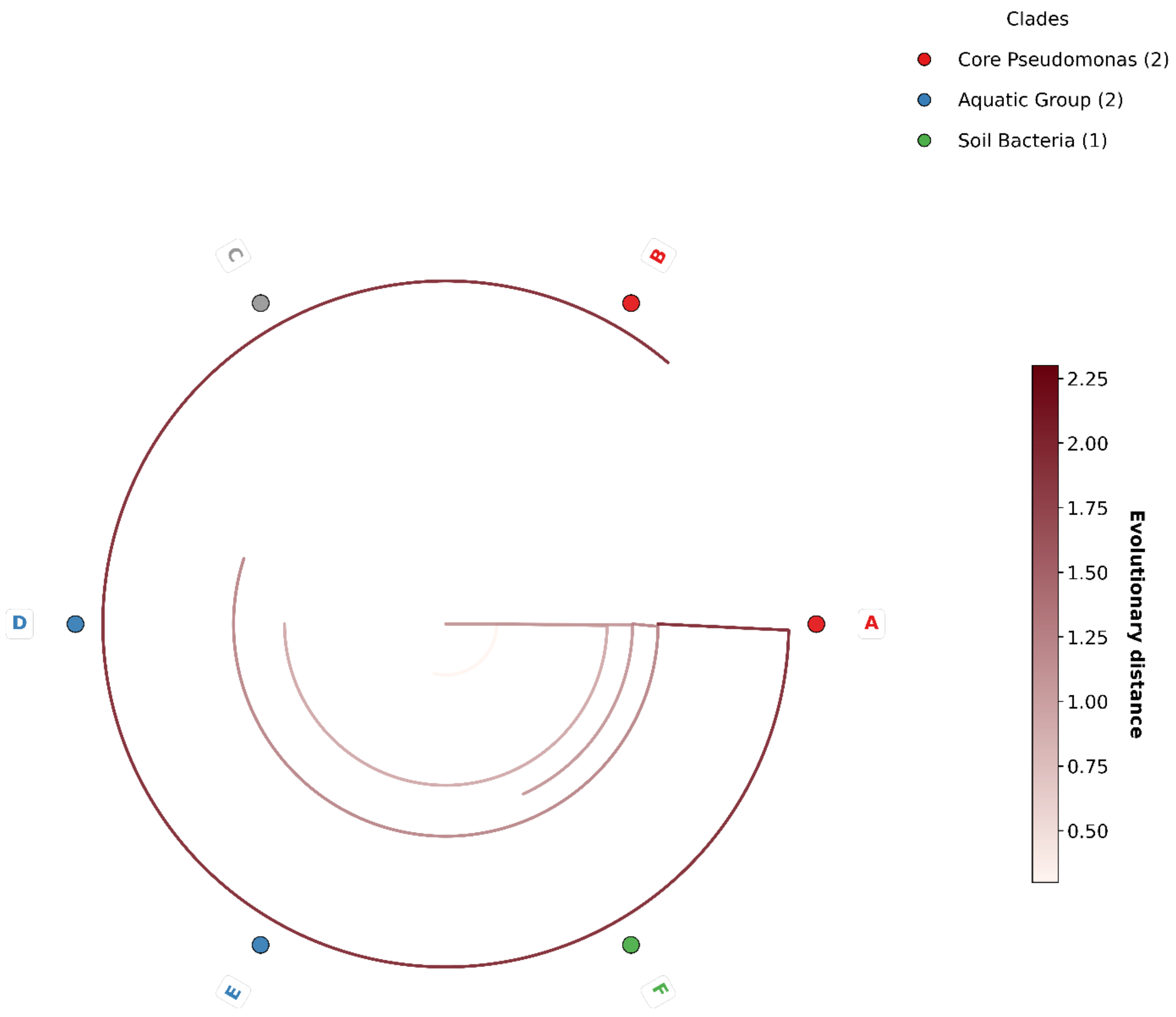

Supplementary Figure S2: Radial phylogenetic tree highlights most common species of sequences extracted from Uniprot database. The phylogenetic tree depicts evolutionary relationships among six bacterial species (A-F), with branch lengths scaled to genetic divergence (measured in evolutionary distance units). Highlighted clades include Core Pseudomonas (species A, B; red), Aquatic Group (species D, E; blue), and Soil Bacteria (species F; green). Branches are colored on a gradient (white-to-red) reflecting divergence magnitude, with terminal nodes marked by colored circles corresponding to their clade. The tree was generated from the Newick string, using average linkage clustering.

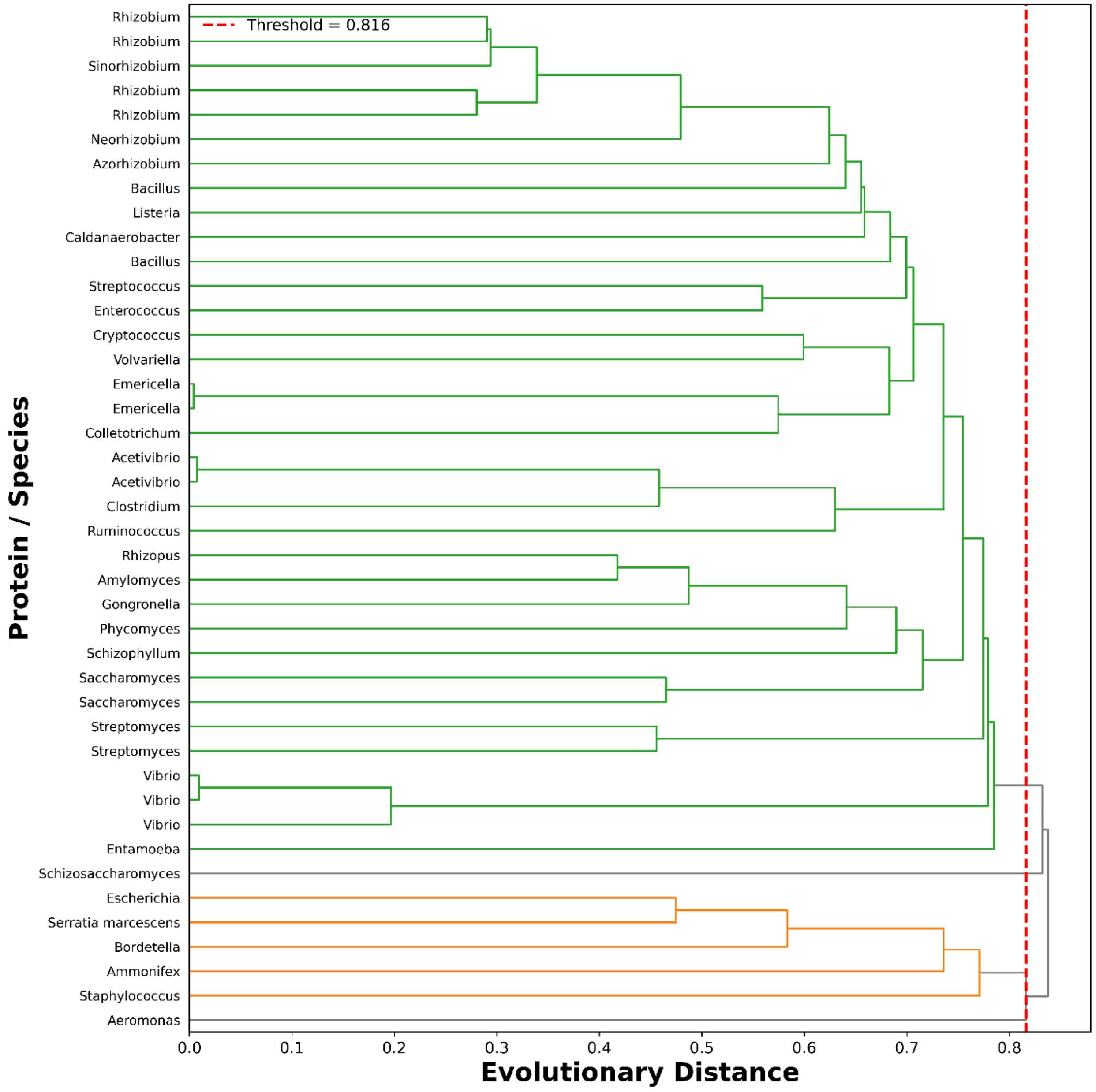

Supplementary Figure S3: Hierarchical clustering dendrogram of microbial species based on CAZy (Carbohydrate-Active enZymes) family annotation. CAZy sequence of characterized enzymes, showed 3 distinct functional clusters (threshold distance = 0.816). Generated from a Newick tree (UPGMA/average linkage), the analysis highlights: Branch lengths are scaled to evolutionary distance (units in substitutions/site). The red dashed line (y=0.816) demarcates clusters. Tight subclustering implies conserved CAZy sequences. The Newick tree was parsed from pairwise distances, clustered via UPGMA (Python/SciPy).

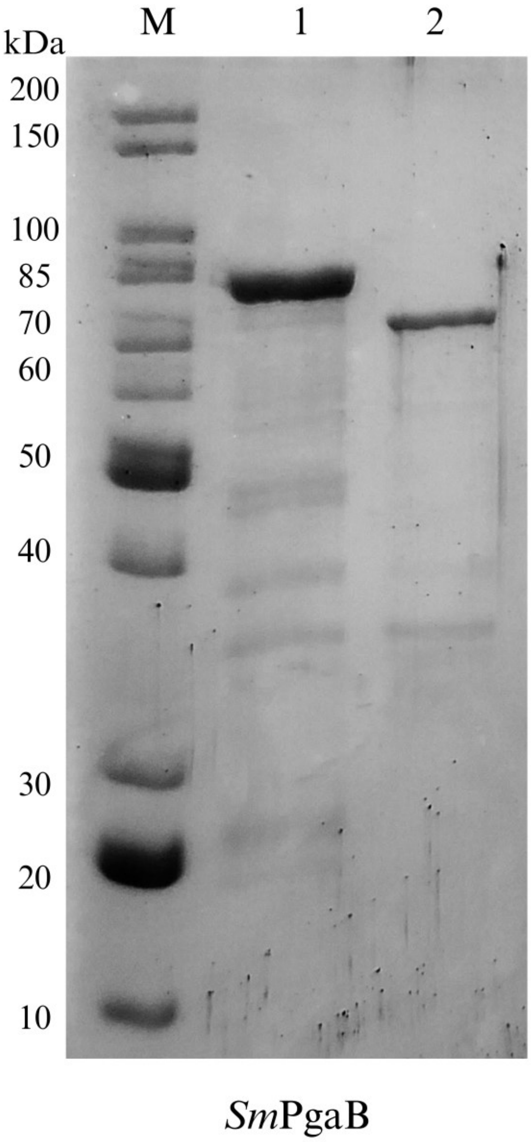

Supplementary Figure S4: Purification process. Lanes: M – molecular-weight standards labeled in kDa, 1 – *Sm*PgaB attached to 6xHis-Trx-tag after the first Ni^{+2} affinity chromatography.

a)

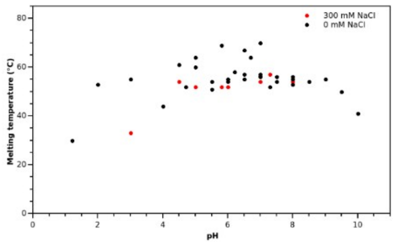

b)

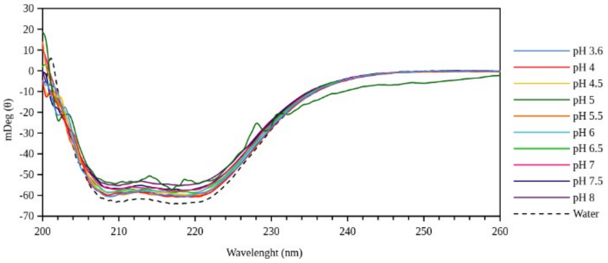

c)

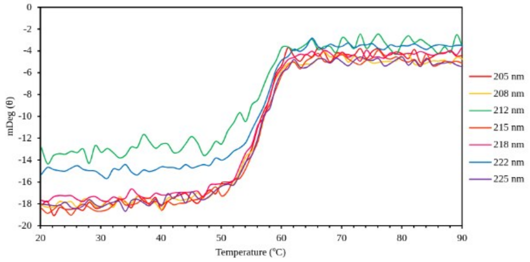

Supplementary Figure S5: a) ThermoFluor assay of *Sm*PgaB using different buffers with pHs from 1.2 to 10; b) Circular dichroism spectrum of *Sm*PgaB in 10 mM buffer with pH range of 3.6-8; c) Thermal denaturation of *Sm*PgaB in 10 mM potassium phosphate buffer pH 6 assessed in the minimum CD wavelengths as a function of temperature.

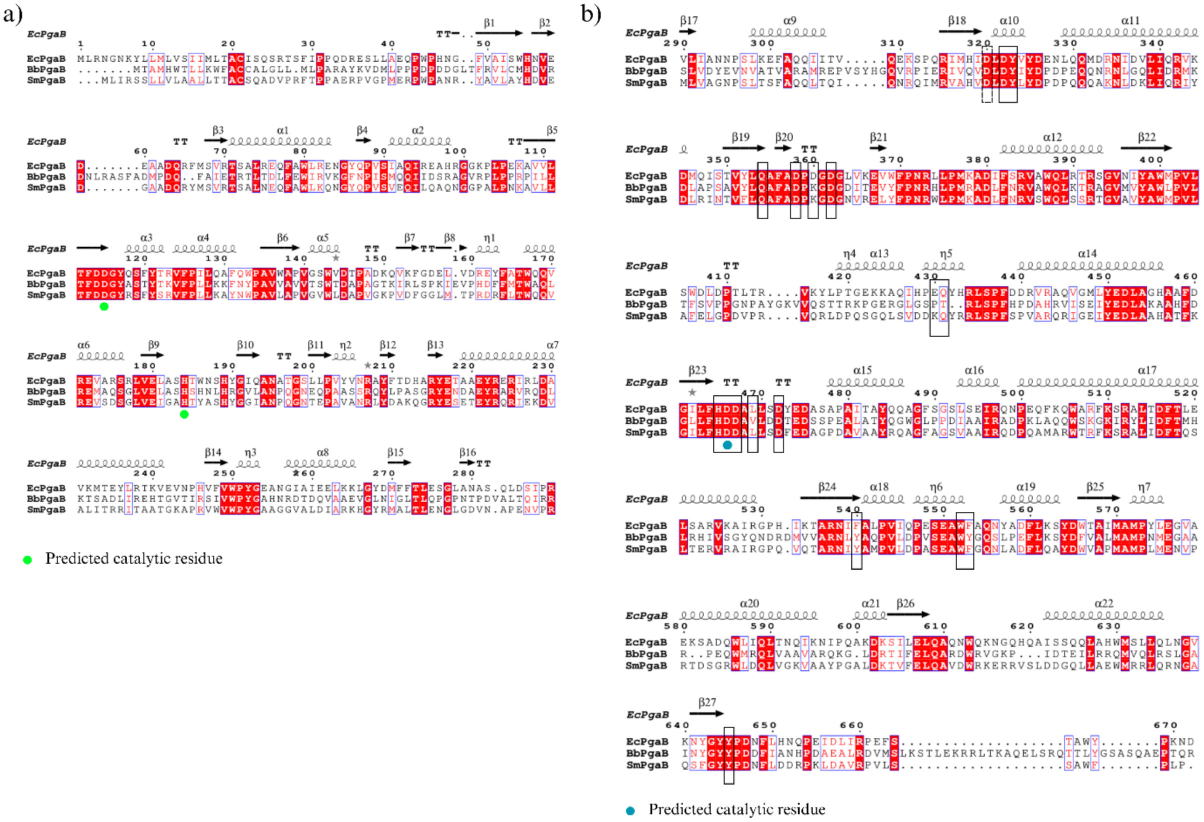

Supplementary Figure S6: a) Alignment of *Sm*PgaB CE4 domain with the orthologues *Ec*PgaB (PDB id: 3VUS) and *Bb*PgaB (PDB id: 5BU6), the highlighted residues are identical or similar in the structures analyzed. The predicted catalytic residues are marked by a green circle; b) Alignment of *Sm*PgaB GH153 domain with the orthologues *Ec*PgaB (PDB id:4P7R) and *Bb*PgaB (PDB id: 6AU1), the highlighted residues are identical or similar in the structures analyzed. The predicted catalytic residue is marked by a blue circle.

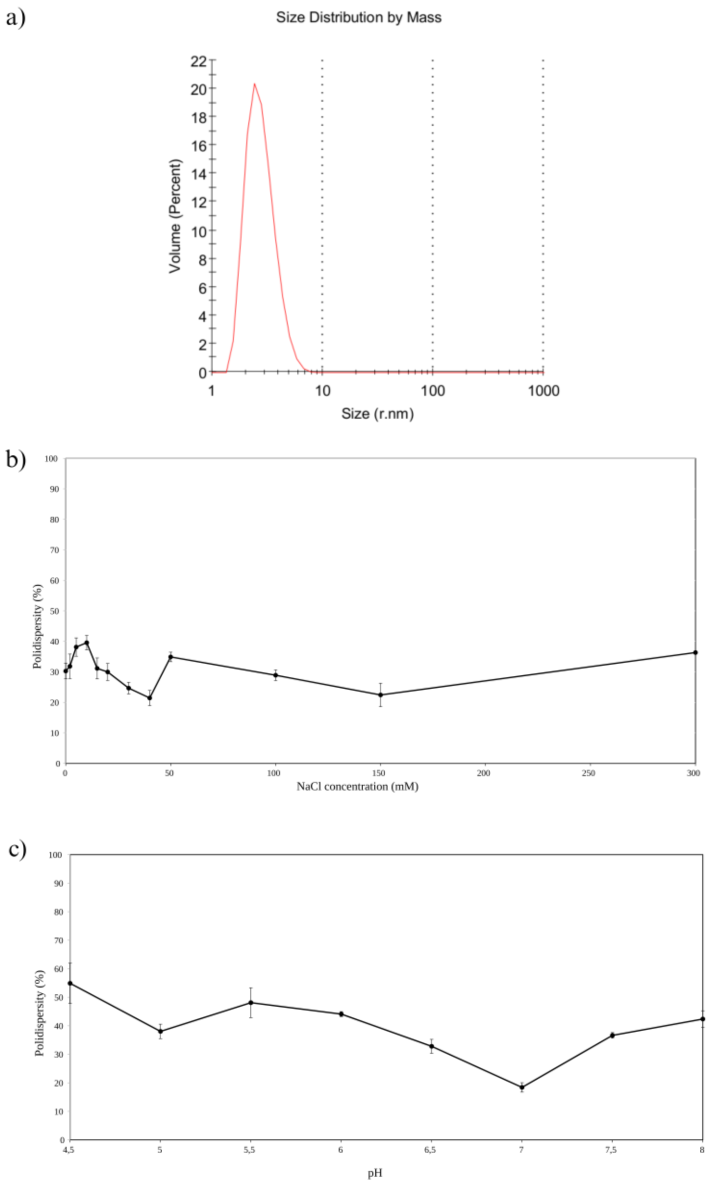

Supplementary Figure S7: Molecular mass size distribution of *Sm*PgaB evaluated by DLS. Polydispersity index as a function of c) NaCl concentration (0-300 mM) and d) pH (4.5-8.0). The enzyme (12 uM) was incubated for 2 h in 10 mM of different buffers prior to assays.

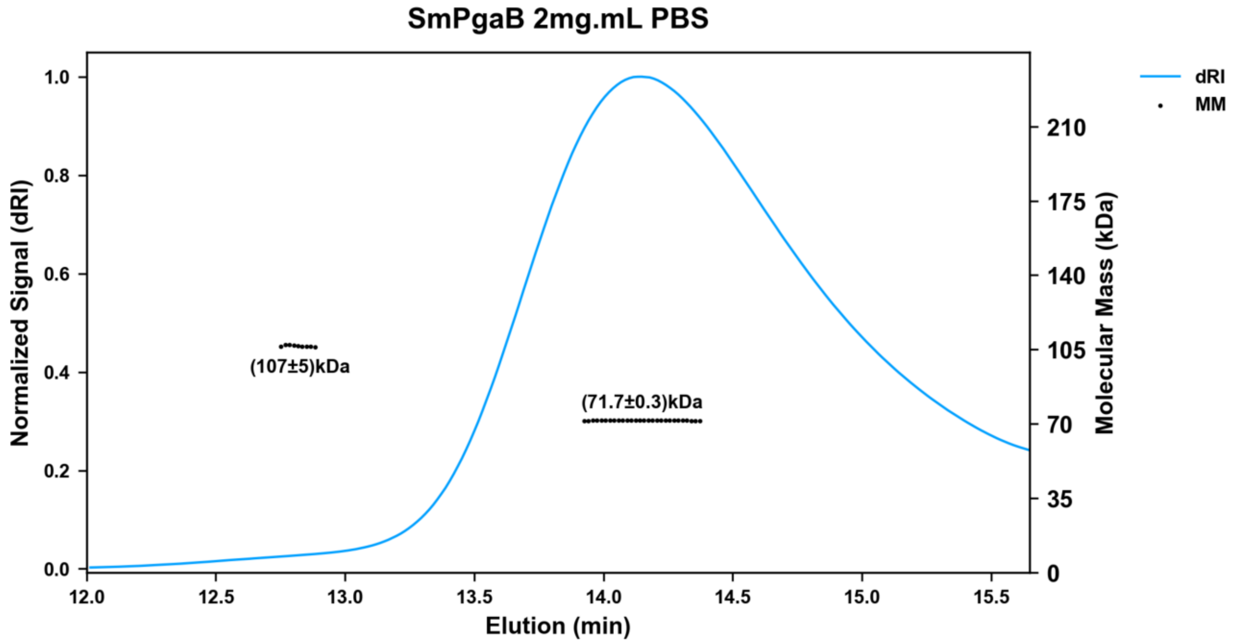

Supplementary Figure S8: SEC-MALS analysis in PBS as a function of the elution time, the molecular weight, and normalized differential refractive index (DRI). a) Elution profile of *Sm*PgaB at 2 mg/mL concentration.

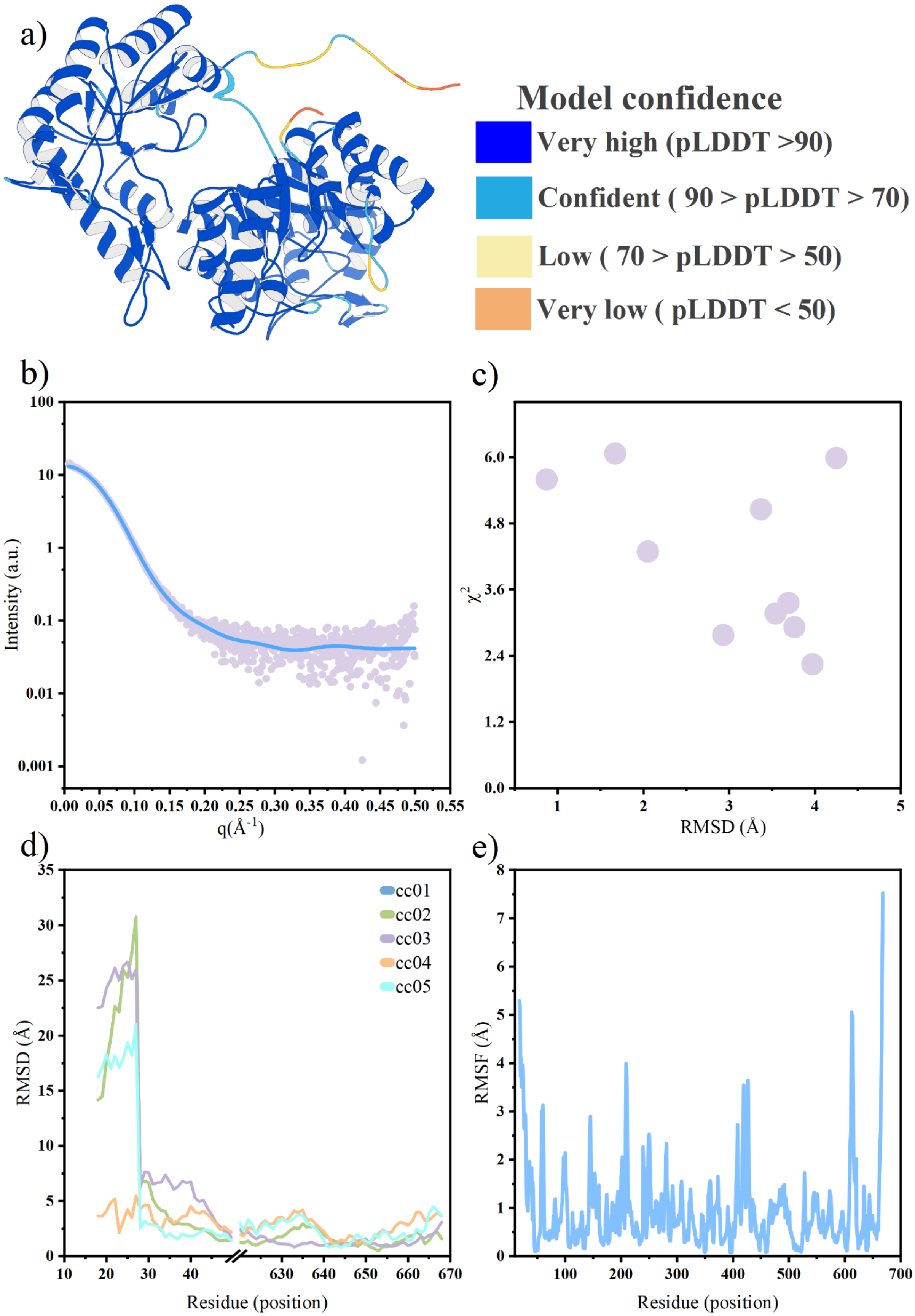
a)
Model confidence
Very high (pLDDT >90)
Confident (90 > pLDDT > 70)
Low (70 > pLDDT > 50)
Very low (pLDDT < 50)
b)
Intensity (a.u.)
q(Å-1)
c)
χ2
RMSD (Å)
d)
RMSD (Å)
cc01
cc02
cc03
cc04
cc05
Residue (position)
e)
RMSF (Å)
Residue (position)

Figure S9: Conformational flexibility analysis of *Sm*PgaB. a) AlphaFold-predicted 3D structure of *Sm*PgaB colored according to model confidence (pLDDT score); b) SAXS experimental scattering curves (purple circles) fitted with theoretical scattering profiles from SREFLEX model; c) Comparison of model deviation (RMSD) and SAXS fitting accuracy (χ^2) from normal mode analysis in SREFLEX; d) Root-mean-square-deviation (RMSD) per residue obtained from SREFLEX simulations; e) Root-mean-square-deviation (RMSD) per residue from CABS-flex simulations.

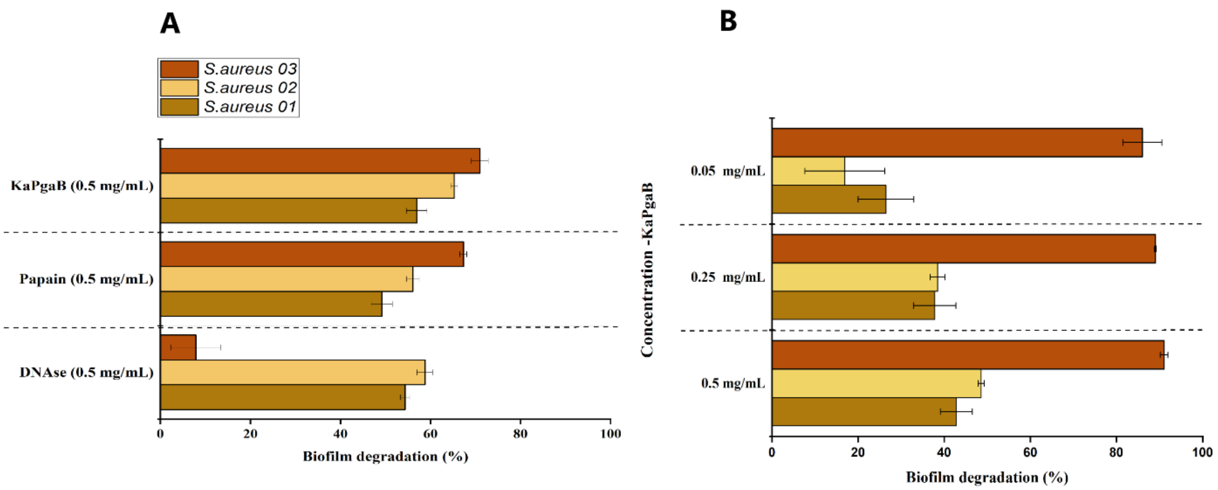

Figure S10: Degradation of *S. aureus* 1, 2 and 3 biofilms using additional enzymes. a) *Klebsiella aerogenes* PgaB, papain and DNAse were used to evaluate the accessibility of PNAG, protein and eDNA fractions of the biofilms. Each enzyme was applied in 0.5mg/mL concentration. b) *S. aureus* 1, 2 and 3 biofilm degradation using different concentrations of *Klebsiella aerogenes* PgaB enzyme, which is known to efficiently hydrolyze PNAG[28]. *S. aureus* 3 biofilm degradation by *Klebsiella aerogenes* PgaB is clearly superior to that of *S. aureus* 1 and 2 biofilms in all tested enzymatic dosages.

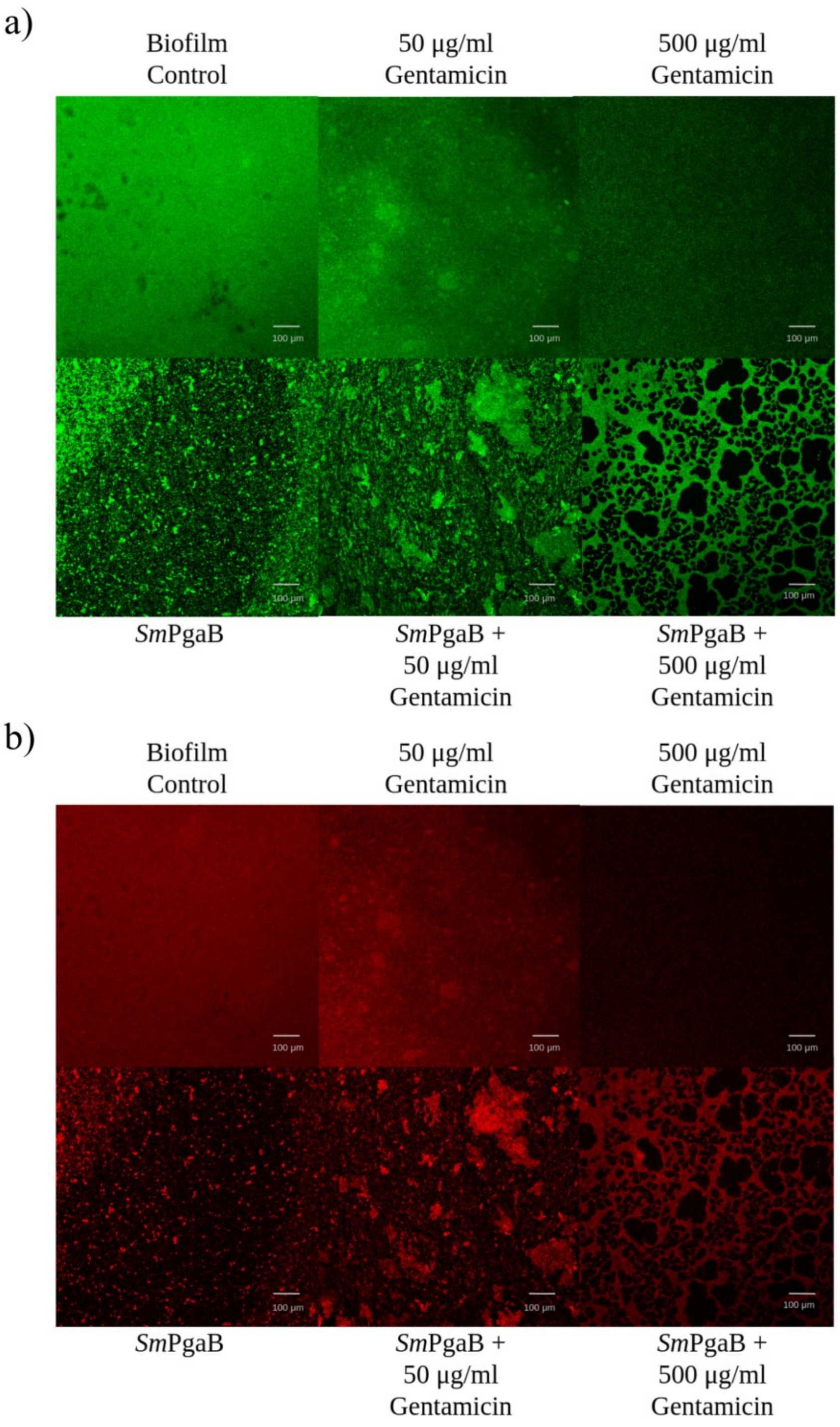

Figure S11: CSLM image of *S. aureus* 3 biofilm stained with LIVE/DEAD stains before and after the treatment with 50 µg/mL *Sm*PgaB and gentamicin. a) Green channel (live cells); b) Red channel (dead cells).

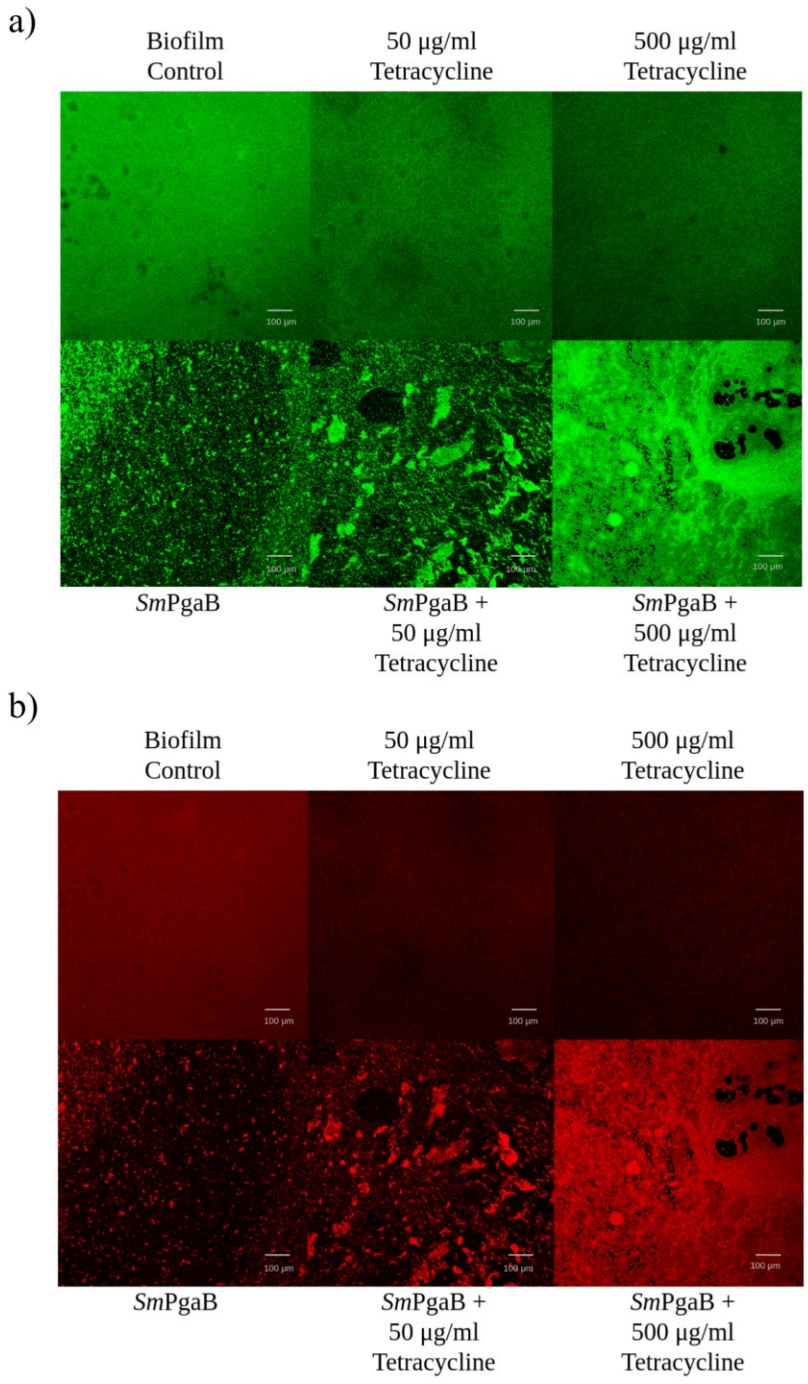

Figure S12: CSLM image of *S. aureus* 3 biofilm stained with LIVE/DEAD stains before and after the treatment with 50 µg/mL *Sm*PgaB and tetracycline. a) Green channel (live cells); b) Red channel (dead cells).

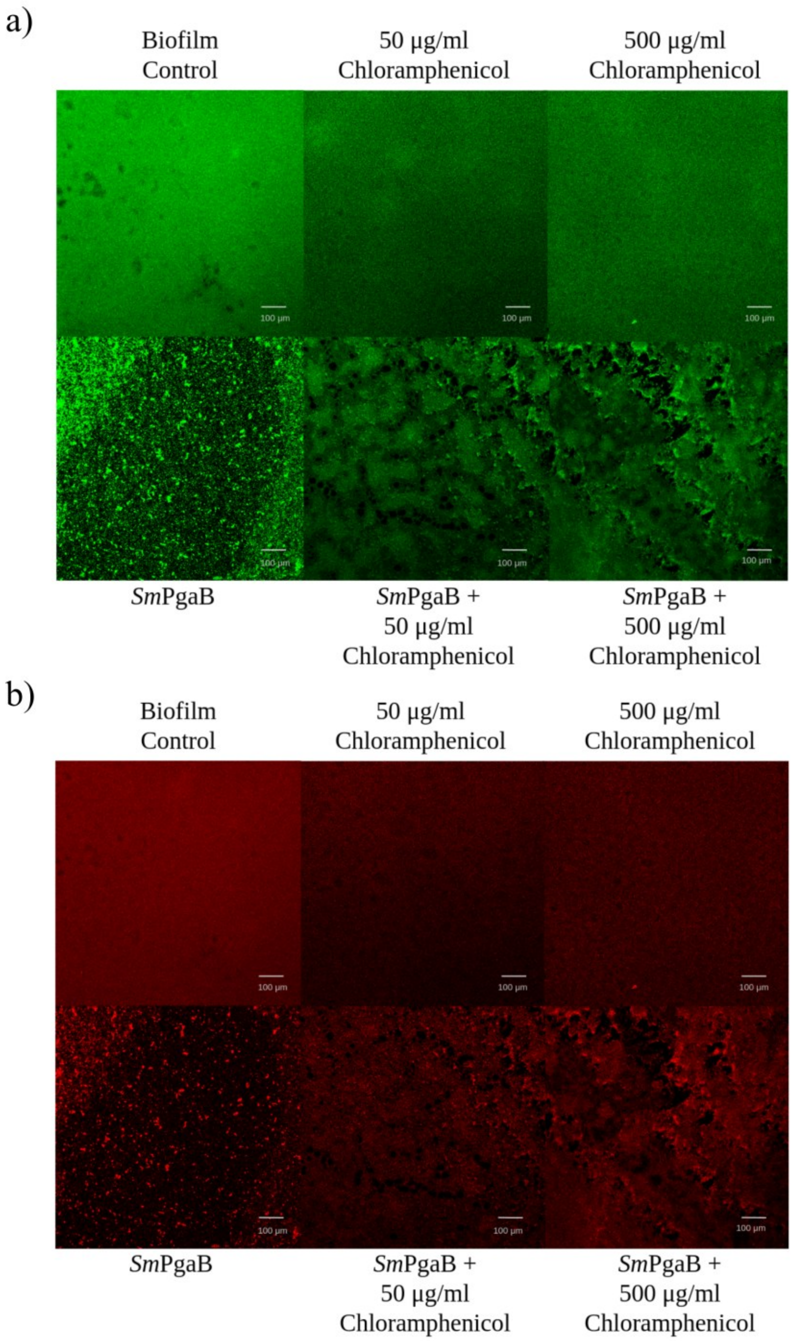

Figure S13: CSLM image of *S. aureus* 3 biofilm stained with LIVE/DEAD stains before and after the treatment with 50 µg/mL *Sm*PgaB and chloramphenicol. a) Green channel (live cells); b) Red channel (dead cells).

Supplementary Tables

Supplementary Table S1 – Sample details, SAXS data collection, analysis, and 3D modelling for *Sm*PgaB.

(*a*) Sample details	
Organism	*Serratia marcescens*
Source (Catalogue No. or reference)	DSM 30121
	*Sm*PgaB
Scattering particle composition	
Protein(s)	A0A379YXX2
Sample environment/configuration	
Solvent composition	10 mM Na_2HPO_4, 1.8 mM KH_2PO_4, 137 mM NaCl, 2.7 mM KCl, pH 7.4
Sample temperature (°C)	20
Size Exclusion Chromatography SEC-SAXS	
Sample injection concentration, mg/ml	8
Sample injection volume, mL	0.05
SEC column type	Superdex 200 Increase PC 3.2/300 (Product#: 28990946)
SEC flowrate, mL/min	0.05
(*b*) SAXS data collection	
Data acquisition/reduction software	Blu-Ice SECSAXS tab/SECPipe
Source/instrument description	BL4-2 at SSRL
Measured *q*-range (q_{min}-q_{max}; Å^{-1})	0.006-0.504
Method for scaling intensities	Arbitrary units (a.u.)
Exposure time(s), number of exposures	0.1, 4
(*c*) SAXS-derived structural parameters	
Methods/Software	
Guinier Analysis	*Sm*PgaB
$I(0) \pm$ s (a.u)	13.28 ± 0.023
R_g ± s (Å)	28.56 ± 0.07
min < qR_g < max	*0.61 < qR_g < 1.29*
PDDF/P(r) analysis	*Sm*PgaB
$I(0) \pm$ s (a.u.)	13.30 ± 0.01
R_g ± s (Å)	28.68 ± 0.04
d_{max} (Å)	94.34

q-range (Å^{-1})	0.0214-0.4664
$P(r)$ fit assessment (definition)	χ^2 1.87
(*d*) Scattering particle size	
Methods/Software	ATSAS and SAXSMoW
	*Sm*PgaB
Volume estimates	
Porod volume, V_p (Å^3)	101681
Molecular weight (M) estimates (kDa)	
From chemical composition	73.292
From SAXS, concentration independent method	69.8
Partial specific volume, n (cm^2/g)	0.7425
Contrast, $\Delta\rho$ (10^{10} cm^{-2})	2.8086
From SAS-independent measure (method)	71.5 ± 0.4 (SEC-MALS)
(*e*) Modelling (a complete sub-panel for each method)	
Shape modelling method(s)	
	*Sm*PgaB
Software	DAMMIF/DAMMIN
q-range for fit (q_{min}-q_{max}, Å^{-1})	0.0214-0.4664
Symmetry/anisotropy assumptions	P1
Number of individual model reconstructions	20
χ^2 for fit	1.92
Cluster Resolution For multiple models (Å)	~18
Software	AlphaFold/Crysol
q-range for fit (q_{min}-q_{max}; Å^{-1})	0.0214-0.4664
Symmetry/anisotropy assumptions	P1
χ^2	5.47
(*f*) Data and model deposition	
SASBDB IDs	SASDXX4

Supplementary References

(1) Edgar, R. C. MUSCLE: Multiple Sequence Alignment with High Accuracy and High Throughput. *Nucleic Acids Res* 2004, *32* (5), 1792–1797. https://doi.org/10.1093/nar/gkh340.

(2) Camilo, C. M.; Polikarpov, I. High-Throughput Cloning, Expression and Purification of Glycoside Hydrolases Using Ligation-Independent Cloning (LIC). *Protein Expr Purif* 2014, *99*, 35–42. https://doi.org/10.1016/j.pep.2014.03.008.

(3) Studier, F. W. Protein Production by Auto-Induction in High Density Shaking Cultures. *Protein Expr Purif* 2005, *41* (1), 207–234. https://doi.org/10.1016/j.pep.2005.01.016.

(4) Berne, R. P. B. J. *Dynamic Light Scattering: With Applications to Chemistry, Biology, and Physics*, illustrate.; Courier Dover Publications, 2000.

(5) Malm, A. V; Corbett, J. C. W. Improved Dynamic Light Scattering Using an Adaptive and Statistically Driven Time Resolved Treatment of Correlation Data. *Sci Rep* 2019, *9* (1), 1–11. https://doi.org/10.1038/s41598-019-50077-4.

(6) Santiago, P. S.; Moura, F.; Moreira, L. M.; Domingues, M. M.; Santos, N. C.; Tabak, M. Dynamic Light Scattering and Optical Absorption Spectroscopy Study of PH and Temperature Stabilities of the Extracellular Hemoglobin of Glossoscolex Paulistus. *Biophys J* 2008, *94* (6), 2228–2240. https://doi.org/10.1529/biophysj.107.116780.

(7) Moysa, A.; Hammerschmid, D.; Szczepanowski, R. H.; Sobott, F.; Dadlez, M. Enhanced Oligomerization of Full-Length RAGE by Synergy of the Interaction of Its Domains. *Sci Rep* 2019, *9* (1), 1–15. https://doi.org/10.1038/s41598-019-56993-9.

(8) Shamir, M.; Amartely, H.; Lebendiker, M.; Friedler, A. Characterization of Protein Oligomers by Multi-Angle Light Scattering. *Encyclopedia of Analytical Chemistry* 2019, 1–17. https://doi.org/10.1002/9780470027318.a9545.

(9) Wyatt, P. J. Light Scattering and the Absolute Characterization of Macromolecules. *Anal Chim Acta* 1993, *272* (1), 1–40. https://doi.org/10.1016/0003-2670(93)80373-S.

(10) Liu, W.; Zhan, C.; Cheng, H.; Kumar, P. R.; Bonanno, J. B.; Nathenson, S. G.; Almo, S. C. Mechanistic Basis for Functional Promiscuity in the TNF and TNF Receptor Superfamilies: Structure of the Light:DcR3 Assembly. *Structure* 2014, *22* (9), 1252–1262. https://doi.org/10.1016/j.str.2014.06.013.

(11) Amartely, H.; Avraham, O.; Friedler, A.; Livnah, O.; Lebendiker, M. Coupling Multi Angle Light Scattering to Ion Exchange Chromatography (IEX-MALS) for Protein Characterization. *Sci Rep* 2018, *8* (1), 1–9. https://doi.org/10.1038/s41598-018-25246-6.

(12) Greenfield, N. J. Using Circular Dichroism Spectra to Estimate Protein Secondary Structure. *Nat Protoc* 2007, *1* (6), 2876–2890. https://doi.org/10.1038/nprot.2006.202.

(13) Micsonai, A.; Wien, F.; Kernya, L.; Lee, Y. H.; Goto, Y.; Réfrégiers, M.; Kardos, J. Accurate Secondary Structure Prediction and Fold Recognition for Circular Dichroism Spectroscopy. *Proc Natl Acad Sci U S A* 2015, *112* (24), E3095–E3103. https://doi.org/10.1073/pnas.1500851112.

(14) Wien, F.; Boros, E.; Vad, H.; Matthieu, R.; Murvai, N.; Lee, Y.; Goto, Y. BeStSel : Webserver for Secondary Structure and Fold Andr as ´ Moln Ar. 2022, *50* (May), 90–98.
(15) Some, D.; Amartely, H.; Tsadok, A.; Lebendiker, M. Characterization of Proteins by Size-Exclusion Chromatography Coupled to Multi-Angle Light Scattering (Sec-Mals). *Journal of Visualized Experiments* 2019, *2019* (148), 1–9. https://doi.org/10.3791/59615.
(16) Matsui, T.; Rajkovic, I.; Mooers, B. H. M.; Liu, P.; Weiss, T. M. Adaptable SEC-SAXS Data Collection for Higher Quality Structure Analysis in Solution. *Protein Science* 2024, *33* (4), 1–16. https://doi.org/10.1002/pro.4946.
(17) Manalastas-Cantos, K.; Konarev, P. V; Hajizadeh, N. R.; Kikhney, A. G.; Petoukhov, M. V; Molodenskiy, D. S.; Panjkovich, A.; Mertens, H. D. T.; Gruzinov, A.; Borges, C.; Jeffries, C. M.; Sverguna, D. I.; Frankea, D. ATSAS 3.0: Expanded Functionality and New Tools for Small-Angle Scattering Data Analysis. *J Appl Crystallogr* 2021, *54*, 343–355. https://doi.org/10.1107/S1600576720013412.
(18) Svergun, D. I. Determination of the Regularization Parameter in Indirect-Transform. *J Appl Crystallogr* 1992, *25*, 495–503.
(19) Franke, D.; Svergun, D. I. DAMMIF, a Program for Rapid Ab-Initio Shape Determination in Small-Angle Scattering. *J Appl Crystallogr* 2009, *42* (2), 342–346. https://doi.org/10.1107/S0021889809000338.
(20) Volkov, V. V; Svergun, D. I. Uniqueness of Ab Initio Shape Determination in Small-Angle Scattering. *J Appl Crystallogr* 2003, *36* (3), 860–864. https://doi.org/10.1107/S0021889803000268.
(21) Svergun, D. I. Restoring Low Resolution Structure of Biological Macromolecules from Solution Scattering Using Simulated Annealing. *Biophys J* 1999, *76* (6), 2879–2886. https://doi.org/10.1016/S0006-3495(99)77443-6.
(22) Kozin, M. B.; Svergun, D. I. Automated Matching of High- and Low-Resolution Structural Models. *J Appl Crystallogr* 2001, *34* (1), 33–41. https://doi.org/10.1107/S0021889800014126.
(23) Piiadov, V.; de Araújo, E. A.; Neto, M. O.; Craievich, A. F.; Polikarpov, I. SAXSMoW 2.0: Online Calculator of the Molecular Weight of Proteins in Dilute Solution from Experimental SAXS Data Measured on a Relative Scale. *Protein Science* 2019, *28* (2), 454–463. https://doi.org/10.1002/pro.3528.
(24) Panjkovich, A.; Svergun, D. I. Deciphering Conformational Transitions of Proteins by Small Angle X-Ray Scattering and Normal Mode Analysis. *Physical Chemistry Chemical Physics* 2016, *18* (8), 5707–5719. https://doi.org/10.1039/c5cp04540a.
(25) Kurcinski, M.; Oleniecki, T.; Ciemny, M. P.; Kuriata, A.; Kolinski, A.; Kmiecik, S. CABS-Flex Standalone: A Simulation Environment for Fast Modeling of Protein Flexibility. *Bioinformatics* 2019, *35* (4), 694–695. https://doi.org/10.1093/bioinformatics/bty685.
(26) Schneidman-Duhovny, D.; Hammel, M.; Sali, A. FoXS: A Web Server for Rapid Computation and Fitting of SAXS Profiles. *Nucleic Acids Res* 2010, *38* (SUPPL. 2), 540–544. https://doi.org/10.1093/nar/gkq461.

(27) Svergun, D.; Barberato, C.; Koch, M. H. CRYSOL - A Program to Evaluate X-Ray Solution Scattering of Biological Macromolecules from Atomic Coordinates. *J Appl Crystallogr* 1995, *28* (6), 768–773. https://doi.org/10.1107/S0021889895007047.

(28) Silva, J.P., Dabul, A.N.G., Rall, V.L.M. et al. Klebsiella aerogenes PgaB orthologue can efficiently hydrolyze Staphylococcus aureus biofilms. World J Microbiol Biotechnol 41, 353 (2025). https://doi.org/10.1007/s11274-025-04550-0
